# Fructose-coated Ångstrom silver prevents sepsis by killing bacteria and attenuating bacterial toxin-induced injuries

**DOI:** 10.7150/thno.55334

**Published:** 2021-07-13

**Authors:** Hao Yin, Mao Zhou, Xia Chen, Teng-Fei Wan, Ling Jin, Shan-Shan Rao, Yi-Juan Tan, Ran Duan, Yu Zhang, Zhen-Xing Wang, Yi-Yi Wang, Ze-Hui He, Ming-Jie Luo, Xiong-Ke Hu, Yang Wang, Wei-Yi Situ, Si-Yuan Tang, Wen-En Liu, Chun-Yuan Chen, Hui Xie

**Affiliations:** 1 Department of Orthopedics, Movement System Injury and Repair Research Center, Xiangya Hospital, Central South University, Changsha, Hunan 410008, China; 2 Angmedicine Research Center of Central South University, Changsha, Hunan 410008, China; 3 Xiangya Hospital of Central South University - Amcan Pharmaceutical Biotechnology Co. Ltd. Joint Research Center, Changsha, Hunan 410008, China; 4 Department of Clinical Laboratory, Xiangya Hospital, Central South University, Changsha, Hunan 410008, China; 5 Xiangya School of Nursing, Central South University, Changsha, Hunan 410013, China; 6 Department of Sports Medicine, Xiangya Hospital, Central South University, Changsha, Hunan 410008, China; 7 Department of Clinical Pharmacology, Xiangya Hospital, Central South University, Changsha, Hunan, China; 8 Institute of Integrative Medicine, Xiangya Hospital, Central South University, Changsha, Hunan 410008, China; 9 Hunan Key Laboratory of Organ Injury, Aging and Regenerative Medicine, Changsha, Hunan 410008, China; 10 Hunan Key Laboratory of Bone Joint Degeneration and Injury, Changsha, Hunan 410008, China; 11 National Clinical Research Center for Geriatric Disorders, Xiangya Hospital, Central South University, Changsha, Hunan 410008, China

**Keywords:** Ångstrom-scale silver particles, bacterial infection, α-hemolysin, lipopolysaccharide, inflammation

## Abstract

Serious infection caused by multi-drug-resistant bacteria is a major threat to human health. Bacteria can invade the host tissue and produce various toxins to damage or kill host cells, which may induce life-threatening sepsis. Here, we aimed to explore whether fructose-coated Ångstrom-scale silver particles (F-AgÅPs), which were prepared by our self-developed evaporation-condensation system and optimized coating approach, could kill bacteria and sequester bacterial toxins to attenuate fatal bacterial infections.

**Methods:** A series of *in vitro* assays were conducted to test the anti-bacterial efficacy of F-AgÅPs, and to investigate whether F-AgÅPs could protect against multi-drug resistant *Staphylococcus aureus* (*S. aureus*)- and *Escherichia coli* (*E. coli*)-induced cell death, and suppress their toxins (*S. aureus* hemolysin and *E. coli* lipopolysaccharide)-induced cell injury or inflammation. The mouse models of cecal ligation and puncture (CLP)- or *E. coli* bloodstream infection-induced lethal sepsis were established to assess whether the intravenous administration of F-AgÅPs could decrease bacterial burden, inhibit inflammation, and improve the survival rates of mice. The levels of silver in urine and feces of mice were examined to evaluate the excretion of F-AgÅPs.

**Results:** F-AgÅPs efficiently killed various bacteria that can cause lethal infections and also competed with host cells to bind with *S. aureus* α-hemolysin, thus blocking its cytotoxic activity. F-AgÅPs inhibited *E. coli* lipopolysaccharide-induced endothelial injury and macrophage inflammation, but not by directly binding to lipopolysaccharide. F-AgÅPs potently reduced bacterial burden, reversed dysregulated inflammation, and enhanced survival in mice with CLP- or *E. coli* bloodstream infection-induced sepsis, either alone or combined with antibiotic therapy. After three times injections within 48 h, 79.18% of F-AgÅPs were excreted *via* feces at the end of the 14-day observation period.

**Conclusion:** This study suggests the prospect of F-AgÅPs as a promising intravenous agent for treating severe bacterial infections.

## Introduction

Overuse and misuse of antibiotics has led to a dramatic increase of bacteria resistant to antibiotics 1, 2. Serious infections (bacteremia, pneumonia, complicated skin infection, *etc*.) caused by antibiotic-resistant bacteria such as methicillin-resistant *Staphylococcus aureus* (*S. aureus*) and multi-drug resistant *Escherichia coli* (*E. coli*) represent a major threat to global public health 2, 3. Bacteria can invade the host tissue and produce exotoxin (α-hemolysin, streptolysin O, pneumolysin, tetanolysin, *etc*.) or endotoxin such as lipopolysaccharide (LPS) to damage or kill host cells. The dysregulated host inflammatory reaction to systemic infection (sepsis) can further cause life-threatening multiple-organ dysfunction 4-6. Antibiotic-resistant bacteria are killing 750 000 people every year 1. If no effective action is taken, the antibiotic-resistant infection-associated deaths are predicted to reach more than 10 million per year by 2050 1. Therefore, there is a pressing need for alternative treatments for severe bacterial infections.

Nanoparticles (NPs) show a promising prospect as new tools to combat deadly bacterial infections due to their distinct physio-chemical traits 7, 8. Silver has been employed as an efficient anti-microbial agent since ancient time and silver NPs (AgNPs) have become one of the most frequently used NPs for anti-bacteria purposes in many industries, such as medical application, textile coatings, food preservation, and cosmetics 9, 10. AgNPs can simultaneously cause bacterial membrane damage and destroy intracellular components to disrupt the normal physiological function of bacteria 9. Besides the anti-bacterial effect, AgNPs are also famous for their anti-inflammatory, pro-wound healing, and anti-cancer activities 10. Recently, we fabricated ultra-small silver particles reaching the Ångstrom (Å, 1 Å = 0.1 nm) scale using a pure physical approach with our self-developed evaporation-condensation system 11. We utilized fructose to disperse and stabilize these particles (AgÅPs) and demonstrated that the intravenous injection of the fructose-coated AgÅPs (F-AgÅPs) could not only suppress pancreatic and lung cancer growth in nude mice, but also inhibit osteosarcoma growth and lung metastasis by altering glucose metabolism in osteosarcoma cells through the inhibition of pyruvate dehydrogenase kinase (PDK) 11, 12. No notable systemic toxicities were detected after F-AgÅPs injection 11, 12. Considering the excellent anti-bacterial and anti-inflammatory properties of silver particles, we hypothesized that F-AgÅPs might have the potential as a safe and efficient intravenous agent for treating serious invasive bacterial infections. Since smaller silver particles have relative larger surface areas and thereby can easily contact with and penetrate bacterial membrane to cause intracellular damage 13, and fructose can be served as a carbon source for many bacteria (e.g., *S. aureus*, *E. coli*, *P. aeruginosa*, and *S. pyogenes*) 14-19 and thereby may facilitate the interaction of F-AgÅPs with bacteria, we supposed that F-AgÅPs might possess a potent anti-bacterial activity superior to the commonly used commercial AgNPs. The ability of F-AgÅPs to affect the activity of intracellular protein in cancer cells prompted us to explore whether F-AgÅPs could sequester bacterial toxins and attenuate bacterial toxins-induced injuries towards host cells.

Here, in order to determine the added benefits of F-AgÅPs over the commercially available AgNPs, we compared the effects of F-AgÅPs with the similarly sized AgNPs on the growth, survival, and structural integrity of various Gram-positive or -negative bacteria that can cause life-threatening invasive infections. Moreover, we evaluated whether F-AgÅPs could protect against multi-drug resistant *S. aureus*- or *E. coli*-induced death of a class of cultured human or mouse cells, and suppress *S. aureus* α-hemolysin-induced cell injury and *E. coli* LPS-induced cell inflammation by directly binding to these toxins. *In vivo*, we investigated whether the intravenous administration of F-AgÅPs was able to attenuate blood and tissue bacterial burdens, reverse the dysregulated inflammatory responses, and improve the survival rates in mice with cecal ligation and puncture (CLP)-induced sepsis and in mouse models of lethal *E. coli* bloodstream infection, either alone or combined with antibiotic therapy.

## Results

### Characterization of AgÅPs and F-AgÅPs

**Figure 1:**
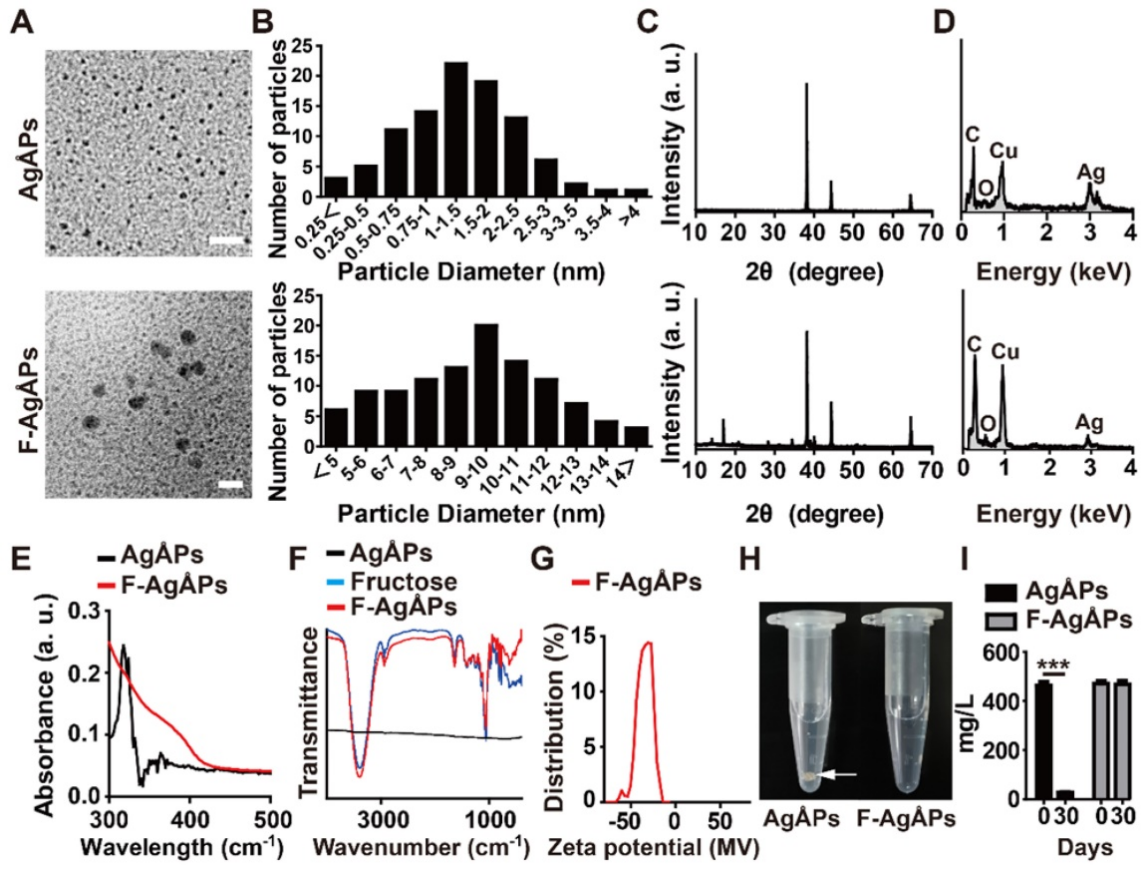
** Characterization of AgÅPs and F-AgÅPs. (A)** Morphologies of AgÅPs and F-AgÅPs detected by TEM. Scale bar: 20 nm. **(B)** Diameters of AgÅPs (14.43 ± 8.14 Ång; *n* = 97) and F-AgÅPs (9.09 ± 3.27 nm; *n* = 107) measured from TEM images. **(C)** XRD patterns of AgÅPs and F-AgÅPs. **(D)** Elemental composition of AgÅPs and F-AgÅPs assessed by EDS. **(E-F)** UV-Vis-NIR (**E**) and FT-IR (**F**) spectra of AgÅPs and F-AgÅPs.** (G)** DLS analysis of the zeta potential of F-AgÅPs. **(H)** Digital photos of AgÅPs and F-AgÅPs in deionized water left for one month at room temperature. White arrow indicates the aggregates formed by AgÅPs. **(I)** ICP-MS analysis of silver concentration in the supernatant of AgÅPs and F-AgÅPs solution at day 0 and day 30. *n* = 3 *per* group. ^***^P < 0.001.

AgÅPs were prepared with our self-designed evaporation-condensation system and fructose was coated on the naked AgÅPs using our recently reported optimized method to obtain F-AgÅPs 12. Consistent with our previous results 12, transmission electron microscope (TEM) images revealed that AgÅPs and F-AgÅPs had sphere-like morphologies with diameters of 14.43 ± 8.14 Ång and 9.09 ± 3.27 nm, respectively (**Figure 1**, **A** and **B**). X-ray diffraction (XRD) patterns of AgÅPs and F-AgÅPs showed strong diffraction peaks at 2*θ* = 38.10°, 44.30° and 64.40° that are corresponding to (111), (200), and (220) planes of face-centered cubic metallic silver, respectively (JCPDS no. 04-0783) (**Figure 1C**). **Figure 1D** shows the elemental composition of AgÅPs and F-AgÅPs by energy dispersive X-ray spectrometry (EDS). F-AgÅPs had a relative lower proportion of silver and higher proportions of carbon and oxygen compared with AgÅPs, indicating the successful coating of fructose on AgÅPs (**Figure 1D**). The incorporation of AgÅPs into fructose was confirmed by ultraviolet-visible near-infrared (UV-Vis-NIR) spectrum analysis, which revealed that AgÅPs, but not F-AgÅPs, had a high intensity peak centered around 318.00 nm (**Figure 1E**), similar to that observed in our previous study 12. Fourier-transform infrared (FT-IR) spectra showed that F-AgÅPs and fructose had similar absorption peaks centered at 3393.62 cm^-1^ and 1059.69 cm^-1^ attributed to the OH and CO stretching vibrations, respectively (**Figure 1F**), which further verified that AgÅPs were successfully coated with fructose. Dynamic light scattering (DLS) analysis showed that the zeta potential of F-AgÅPs in deionized water ranged from -14.15 to -63.46 mV (**Figure 1G**), indicating a good electrostatic stability of F-AgÅPs. After being left at room temperature for 30 days, large aggregates were formed by AgÅPs in deionized water and the silver concentration in the supernatant was markedly decreased, as revealed by the photographs of AgÅPs solution (**Figure 1H**) and silver measurement by inductively coupled plasma mass spectrometry (ICP-MS; **Figure 1I**). However, the solution of F-AgÅPs remained clear and no obvious reduction of silver concentration was observed in the supernatant of F-AgÅPs suspension at days 30 than that at day 0 (**Figure 1**, **H** and **I**), indicating that F-AgÅPs can be stable and well dispersed in aqueous solution.

### Effects of F-AgÅPs on bacterial growth, survival, and structural integrity

**Figure 2:**
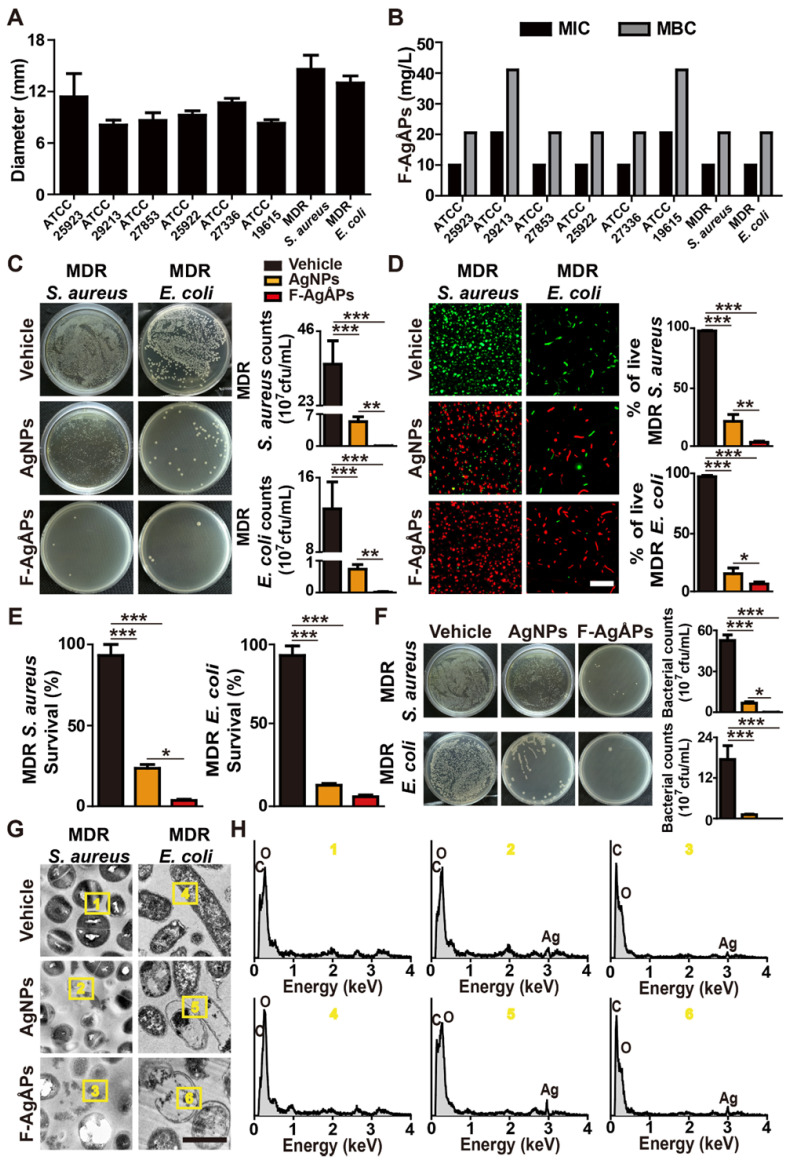
** Effects of F-AgÅPs on bacterial growth, survival, and structural integrity**. **(A)** Diameters of inhibition zones around the paper disks infiltrated with vehicle (normal saline) or F-AgÅPs. *n* = 3 *per* group. MDR: multi-drug-resistant. **(B)** MIC and MBC values of F-AgÅPs against different bacteria. *n* = 3 *per* group.** (C)** Images of bacterial colonies on agar plates formed by the vehicle (normal saline)-, F-AgÅPs-, or AgNPs-treated multi-drug-resistant *S. aureus* or *E. coli* in LB medium. Bacterial colony numbers were counted. *n* = 3 *per* group. **(D)** Calcein-AM/PI staining images of the vehicle (normal saline)-, F-AgÅPs-, or AgNPs-treated multi-drug-resistant *S. aureus* or *E. coli* and quantification of the percentage of live (calcein-AM^+^PI^-^) bacteria. Scale bar: 10 μm. *n* = 3 *per* group. **(E)** Survival rate of bacteria assessed by alamar blue assay. *n* = 4 *per* group. **(F)** Images of bacterial colonies formed by the vehicle (normal saline)-, F-AgÅPs-, or AgNPs-treated multi-drug-resistant *S. aureus* or *E. coli* in mouse blood and quantification of the numbers of bacterial colonies. *n* = 3 *per* group. **(G-H)** Morphological features (**G**) and in situ elemental composition analysis (**H**) of the vehicle (normal saline)-, F-AgÅPs-, or AgNPs-treated multi-drug-resistant *S. aureus* or *E. coli* by TEM combined with EDS. Scale bar: 500 nm. ^***^P < 0.001.

We next evaluated the anti-bacterial effects of F-AgÅPs on a class of Gram-positive or -negative bacteria that pose serious threats to human health, including methicillin-sensitive *S. aureus* ATCC25923, methicillin-resistant *S. aureus* ATCC29213, *Pseudomonas aeruginosa* (*P. aeruginosa*) ATCC27853, *E. coli* ATCC25922, *Streptococcus pneumoniae* (*S. pneumoniae*) ATCC27336, *Streptococcus pyogenes* (*S. pyogenes*) ATCC19615, one strain of clinically isolated multi-drug-resistant *S. aureus*, and one strain of multi-drug-resistant extended-spectrum beta-lactamase (ESBL)-producing *E. coli*. **Figure 2A** shows the diameters of bacterial growth inhibition zones around the filter paper disks soaked with undiluted F-AgÅPs (500 ng μL^-1^) or an equal volume of vehicle (normal saline). The inhibition zone diameters ranged from 8.08 ± 0.57 to 14.57 ± 1.65 mm, depending on the sensitivity of the tested bacteria (**Figure 2A**). The largest inhibition zone sizes (14.57 ± 1.65 mm) were observed in the clinically isolated multi-drug-resistant* S. aureus* treated with F-AgÅPs (**Figure 2A**), indicating the highest susceptibility of this bacterium against F-AgÅPs. The two smallest inhibition zone sizes were observed in the F-AgÅPs-treated methicillin-resistant *S. aureus* ATCC29213 (8.08 ± 0.57 mm) and *S. pyogenes* ATCC19615 (8.28 ± 0.41 mm), indicating the lower sensitivity of these two bacteria against F-AgÅPs compared with other bacteria. Serial dilutions of F-AgÅPs were made and added to the cultures of the above tested bacteria, in order to assess the minimum inhibitory concentration (MIC) and minimum bactericidal concentration (MBC) of F-AgÅPs against these bacteria. Consistently, the higher MIC and MBC values of F-AgÅPs against *S. aureus* ATCC29213 and *S. pyogenes* ATCC19615 compared with other bacteria also revealed the lower sensitivity of these two bacteria to the toxicity of F-AgÅPs (**Figure 2B**).

Subsequently, the anti-bacterial activities of F-AgÅPs at the concentrations of MIC values were further determined in the clinically isolated multi-drug-resistant strains of *S. aureus* and *E. coli*. Meanwhile, the anti-bacterial effects of the commercial similarly sized AgNPs at the same concentration were also assessed. The tested bacteria were pretreated with F-AgÅPs, AgNPs, or vehicle for 8 h and a small volume of bacterial solution was spread onto a Luria-Bertani (LB) agar plate. As shown in **Figure 2C**, large numbers of bacterial colonies were formed on agar plates by the vehicle-treated bacteria, while only a few bacterial colonies were observed in F-AgÅPs treatment group, suggesting the potent inhibitory effect of F-AgÅPs on bacterial survival or/and proliferation. AgNPs could also inhibit bacterial colony formation on LB agar plate, but the effect was much lower than that of F-AgÅPs (**Figure 2C**). Multi-drug-resistant *S. aureus* and *E. coli* were then incubated with F-AgÅPs, AgNPs, or vehicle in LB medium for 3 h and subjected to calcein-AM/propidium iodide (PI) staining. The result demonstrated that treatment with F-AgÅPs caused a higher extent of reduction in the numbers of calcein-AM-positive and PI-negative (calcein-AM^+^PI^-^) live *S. aureus* and *E. coli* compared with the AgNPs group (**Figure 2D**). The higher ability of F-AgÅPs than AgNPs to inhibit the survival of these bacteria was further confirmed by the alamar blue assay (**Figure 2E**). To mimic the *in vivo* bloodstream infection, *S. aureus* and *E. coli* were inoculated into the isolated blood from mice and treated with F-AgÅPs, AgNPs, or vehicle for 3 h. Bacterial colony counting assay showed a marked decrease of bacterial colony numbers in F-AgÅPs and AgNPs treatment groups compared to the vehicle-treated control group (**Figure 2F**), suggesting the potential of F-AgÅPs and AgNPs to inhibit *S. aureus* and *E. coli* bloodstream infections. However, the ability of AgNPs to reduce blood bacterial loads was much weaker than F-AgÅPs (**Figure 2F**). TEM observations revealed that the vehicle-treated *S. aureus* and *E. coli* exhibited the normal spherical- or rod-shaped morphology with intact cell wall and membrane (**Figure 2G**). When the bacteria were treated with F-AgÅPs or AgNPs, the deformation of bacterial structure and damage of cell wall and membrane were observed, whereas F-AgÅPs caused much more dramatic damage to bacterial structural integrity, resulting in the lysis of cells and leakage of intracellular contents (**Figure 2G**). The entry of F-AgÅPs and AgNPs into bacteria was confirmed by the presence of elemental silver in the F-AgÅPs- or AgNPs-treated bacteria by TEM combined with EDS analysis (**Figure 2H**). These findings demonstrate that F-AgÅPs possess superior capacities than the commercial similarly sized AgNPs to inhibit the growth and survival of bacteria and destroy the structural integrity of bacteria.

### F-AgÅPs attenuate bacteria or bacterial toxins-induced cell injuries

**Figure 3:**
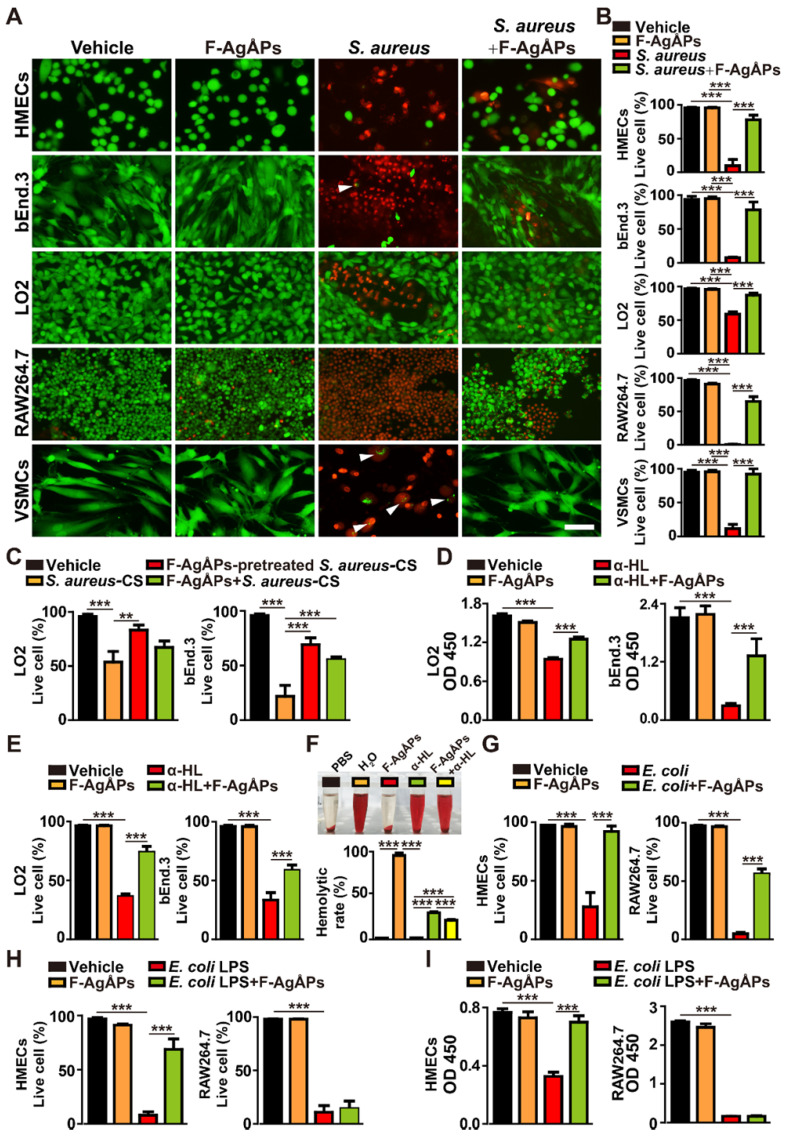
** F-AgÅPs attenuate bacteria or bacterial toxins-induced cell injuries. (A)** Calcein-AM/PI staining images of a class of human or mouse cells treated with vehicle, F-AgÅPs, *S. aureus*, or *S. aureus* + F-AgÅPs. Vehicle indicates normal saline (solvent of F-AgÅPs) + un-cultured medium (for culturing *S. aureus*). White arrows indicate live bacteria-like signals inside or around the dead recipient cells. Scale bar: 100 μm. **(B)** The ratios of calcein-AM^+^PI^-^ live cells in (**A**). *n* = 3 *per* group. **(C)** The ratios of calcein-AM^+^PI^-^ live LO2 and bEnd.3 cells in vehicle, *S. aureus*-CS, F-AgÅPs*-*pretreated* S. aureus*-CS, and F-AgÅPs + *S. aureus*-CS treatment groups. *S. aureus*-CS:* S. aureus*-derived culture supernatant. Vehicle indicates normal saline (solvent of F-AgÅPs) + un-cultured medium (for culturing *S. aureus*). *n* = 3 *per* group. **(D)** CCK-8 analysis of cell survival/growth in vehicle, F-AgÅPs, α-HL, and α-HL + F-AgÅPs treatment groups. α-HL: α-hemolysin. Vehicle indicates normal saline (solvent of F-AgÅPs) + PBS (solvent of α-HL).* n* = 4 *per* group. **(E)** The ratios of calcein-AM^+^PI^-^ live cells in vehicle (normal saline + PBS), F-AgÅPs, α-HL, and α-HL + F-AgÅPs treatment groups. *n* = 3 *per* group. **(F)** Digital photos of RBC suspension and the hemolytic rates based on the relative absorbance of free hemoglobin at 541 nm. RBC treated with water (H_2_O: positive control) or PBS (negative control) served as controls. *n* = 3 *per* group. **(G)** The ratios of calcein-AM^+^PI^-^ live cells in vehicle, F-AgÅPs, *E. coli*, and *E. coli* + F-AgÅPs treatment groups. Vehicle indicates normal saline (solvent of F-AgÅPs) + PBS (solvent of LPS). *n* = 3 *per* group. **(H)** The ratios of calcein-AM^+^PI^-^ live cells in vehicle (normal saline + PBS), F-AgÅPs, *E. coli* LPS, and *E. coli* LPS + F-AgÅPs treatment groups. *n* = 3 *per* group. **(I)** CCK-8 analysis of cell survival/growth in different treatment groups. *n* = 4 *per* group. ^**^P < 0.01, ^***^P < 0.001.

To explore whether F-AgÅPs could directly protect host cells from *S. aureus*-induced injuries, human microvascular endothelial cells (HMECs), mouse brain endothelial cells bEnd.3, human hepatocytes (LO2), mouse macrophage cells RAW264.7, and human vascular smooth muscle cells (VSMCs) were incubated with multi-drug-resistant *S. aureus* and F-AgÅPs or vehicle for 6 h. Calcein-AM/PI staining showed that treatment with *S. aureus* markedly reduced the percentages of live (calcein-AM^+^PI^-^) cells, especially RAW264.7, but co-incubation with F-AgÅPs significantly increased the survival of these *S. aureus*-treated cells (**Figure 3**, **A** and **B**). No obvious differences were detected in the ratios of live cells between the vehicle- and F-AgÅPs-treated cells (**Figure 3**, **A** and **B**), suggesting that F-AgÅPs at a therapeutically effective dose have no notable toxicity towards these healthy cells. Consistently, incubation with the culture supernatant of *S. aureus* (*S. aureus*-CS) significantly reduced the viability of LO2 and bEnd.3 cells, whereas co-treatment with *S. aureus*-CS and F-AgÅPs, or treatment with the F-AgÅPs-pretreated *S. aureus*-CS, induced lower levels of cell death, as revealed by the changes of the percentages of calcein-AM^+^PI^-^ live cells (**Figure 3C**), suggesting the efficacy of F-AgÅPs against the cell injury induced by the secreted factors from *S. aureus*. α-hemolysin, one of the exotoxins produced by *S. aureus*, is a significant virulence factor that plays an important role in the pathogenesis of *S. aureus* infections 20.

Cell counting kit-8 (CCK-8) assay revealed that direct incubation of LO2 and bEnd.3 with α-hemolysin significantly inhibited the survival/growth of these cells, whereas the effect was notably reversed by co-treatment with F-AgÅPs (**Figure 3D**). The protective action of F-AgÅPs against the α-hemolysin-induced toxicity was further demonstrated by calcein-AM/PI staining, which showed higher percentages of calcein-AM^+^PI^-^ live cells in α-hemolysin + F-AgÅPs group compared with α-hemolysin group (**Figure 3E**). Digital photos of red blood cell (RBC) suspension and the hemolytic rates based on the relative absorbance of free hemoglobin at 541 nm indicated the hemolytic activity of α-hemolysin, which caused 31.69 % ± 1.07 % of RBC lysis (**Figure 3F**). F-AgÅPs did not show any hemolytic effect and there was a statistically significant reduction of lysis of RBC co-treated with F-AgÅPs and α-hemolysin (**Figure 3F**). These results suggest that F-AgÅPs may have the potential to directly interact with α-hemolysin to block its cytotoxicity.

We also investigated whether F-AgÅPs could prevent *E. coli*- and *E. coli* LPS-induced cell injuries. Calcein-AM/PI staining revealed that both *E. coli* and *E. coli*-derived LPS were capable of reducing the percentages of live (calcein-AM^+^PI^-^) HMECs and RAW264.7 cells (**Figure 3**, **G** and **H**). Co-incubation with F-AgÅPs could remarkably enhance the survival of the *E. coli*- or *E. coli* LPS-treated HMECs and the *E. coli*-treated RAW264.7, but had no protective effect on the viability of the *E. coli* LPS-treated RAW264.7 cells (**Figure 3**, **G** and **H**). CCK-8 assay also showed that F-AgÅPs could block the *E. coli* LPS-induced reduction of survival/growth of HMECs, but not RAW264.7 (**Figure 3I**). These results suggest that the protective effects of F-AgÅPs against the cytotoxicity of *E. coli* in endothelial cells may be mediated by inhibiting LPS-induced damage, but the blockade of LPS is not the primary mechanism by which F-AgÅPs prevent the *E. coli*-induced injury of macrophage cells.

### F-AgÅPs bind to *S. aureus* α-hemolysin to inhibit its activity and down-regulate LPS-induced macrophage inflammation

**Figure 4:**
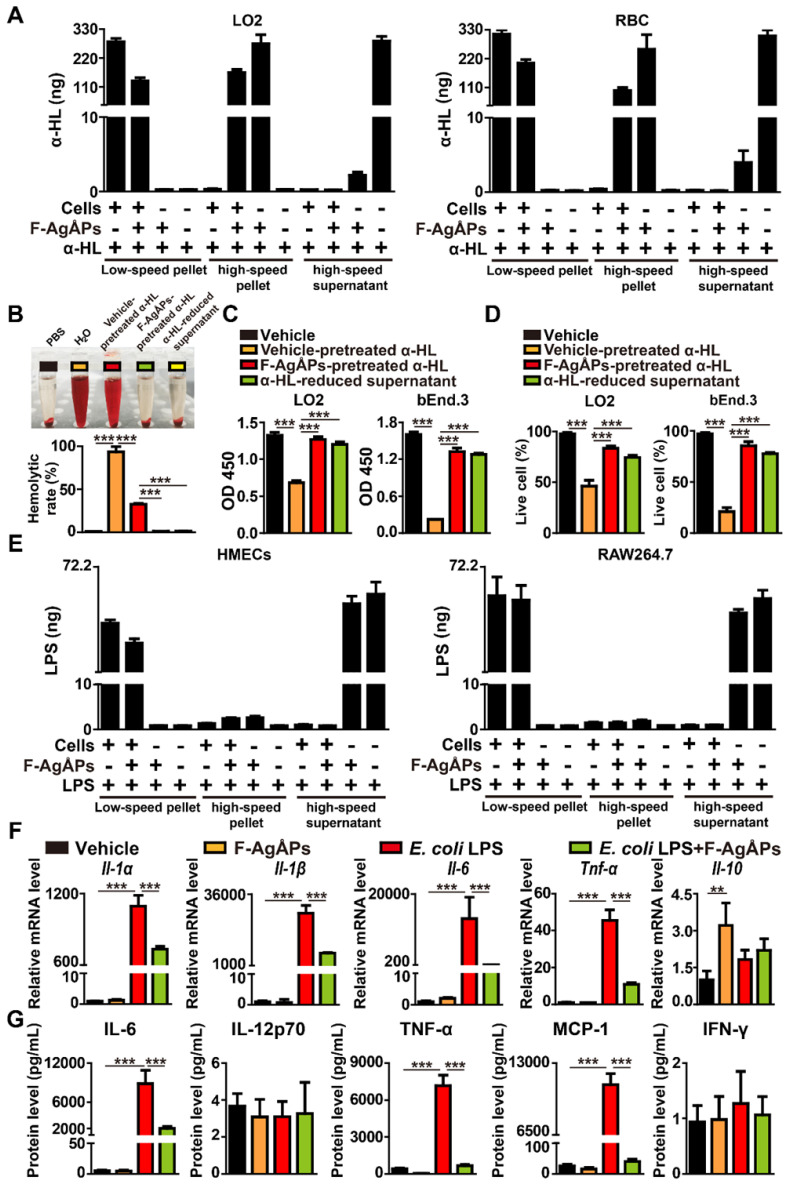
** F-AgÅPs bind to *S. aureus* α-hemolysin to inhibit its activity and down-regulate LPS-induced macrophage inflammation. (A)** Analysis of α-hemolysin content by ELISA. *n* = 3 *per* group. **(B)** Gross observation and the hemolytic rates in RBC treated with PBS, H_2_O, vehicle (normal saline) or F-AgÅPs-pretreated α-HL, or α-HL-reduced supernatant. α-HL: α-hemolysin. *n* = 3 *per* group. **(C)** CCK-8 analysis of cell survival/growth in vehicle (normal saline + PBS), vehicle (normal saline)- or F-AgÅPs-pretreated α-HL, and α-HL-reduced supernatant treatment groups. *n* = 4 *per* group. **(D)** The ratios of calcein-AM^+^PI^-^ live cells in different treatment groups. *n* = 3 *per* group. **(E)** Analysis of *E. coli* LPS content by ELISA. *n* = 3 *per* group. **(F)** qRT-PCR analysis of *Il-1α*, *Il-1β*, *Il-6*, *Tnf-α*, and *Il-10* gene expression in RAW264.7 macrophages treated with vehicle, F-AgÅPs, *E. coli* LPS, or *E. coli* LPS + F-AgÅPs. Vehicle indicates normal saline (solvent of F-AgÅPs) + PBS (solvent of LPS). *n* = 3 *per* group. **(G)** Protein levels of IL-6, IL-12p70, TNF-α, MCP-1, and IFN-γ measured by a CBA inflammation kit combined with flow cytometry. *n* = 3 *per* group. ^***^P < 0.001.

To determine whether F-AgÅPs could bind to *S. aureus* α-hemolysin, α-hemolysin was added to different cell types (LO2 and RBC) with or without F-AgÅPs, to F-AgÅPs without cells, or to vehicle only. After differential centrifugation of these samples, the low-speed pellet (cells), high-speed pellet (F-AgÅPs), and soluble fraction (free α-hemolysin) were obtained and subjected to enzyme-linked immunosorbent assay (ELISA) for testing α-hemolysin content. As shown in **Figure 4A**, a much higher level of α-hemolysin was detected in low-speed pellet from cells + α-hemolysin group and cells + α-hemolysin + F-AgÅPs group, high-speed pellet from cells + α-hemolysin + F-AgÅPs group and especially α-hemolysin + F-AgÅPs group, and high-speed supernatant fraction from α-hemolysin group, but not in pellet or supernatant from other groups, suggesting that F-AgÅPs have the ability to sequester *S. aureus* α-hemolysin, thus resulting in a decreased amount of hemolysin to bind to human or mouse cells. A substantial level of α-hemolysin detected in low-speed pellet from cells + α-hemolysin + F-AgÅPs group (**Figure 4A**) might be due to the uptake of the α-hemolysin-binding F-AgÅPs or/and the residual free α-hemolysin by the recipient cells.

We then assessed whether binding with F-AgÅPs could affect the cytotoxic activities of α-hemolysin. The bacterial toxin was pretreated with F-AgÅPs for 30 min, followed by high-speed centrifugation to obtain the pellet (F-AgÅPs binding with α-hemolysin) and the α-hemolysin-reduced supernatant. To exclude the possibility of the procedures of high-speed centrifugation on the function of this bacterial toxin, α-hemolysin pretreated with the vehicle of F-AgÅPs (normal saline) was also subjected to high-speed centrifugation and the supernatant with abundant free α-hemolysin was harvested. The vehicle-pretreated α-hemolysin in the supernatant was used as a control to determine whether the F-AgÅPs-pretreated α-hemolysin lost its cytotoxic activities. Unlike the free α-hemolysin, the F-AgÅPs-pretreated α-hemolysin failed to induce RBC lysis, as revealed by the clear and transparent appearance of RBC suspension and the much lower hemolytic rates (1.61% ± 0.02%) compared to that of the vehicle-pretreated α-hemolysin-treated cells (34.95% ± 0.98%) (**Figure 4B**). CCK-8 assay showed that only the vehicle-pretreated α-hemolysin, but not the F-AgÅPs-pretreated α-hemolysin, reduced the survival/growth of LO2 and bEnd.3 cells (**Figure 4C**). Consistently, calcein-AM/PI staining of live and dead cells indicated that pre-treatment with F-AgÅPs significantly blocked the ability of α-hemolysin to kill these cells (**Figure 4D**). As expected, the hemolysis assays, CCK-8 assay, and calcein-AM/PI staining demonstrated that the α-hemolysin-reduced supernatant had no significant hemolytic activity and cytotoxic effects on LO2 and bEnd.3 cells (**Figure 4B-D**). These results indicate that the sequestration of α-hemolysin by F-AgÅPs can block the functional activity of α-hemolysin.

We also determined whether F-AgÅPs could sequester *E. coli* LPS using the similar method that was used to verify the binding relationship between F-AgÅPs and *S. aureus* α-hemolysin. As evidenced by ELISA, a much higher level of LPS was detected in low-speed pellet from cells + LPS group and cells + LPS + F-AgÅPs group, and high-speed supernatant from LPS + F-AgÅPs group and LPS group (**Figure 4E**). Although the high-speed pellet from cells + LPS + F-AgÅPs group and LPS + F-AgÅPs group showed a higher amount of LPS compared with high-speed pellet from cells + LPS group and/or LPS group, the increase of LPS remained very low (**Figure 4E**). These findings suggest no preferential binding between F-AgÅPs and *E. coli* LPS, even if F-AgÅPs are capable of protecting against *E. coli* LPS-induced injuries towards HMECs.

LPS is a potent stimulus that triggers inflammatory responses in many cell types such as macrophages in the host 21, 22. To test whether F-AgÅPs could affect LPS-induced inflammation, F-AgÅPs or vehicle was added to the cultures of RAW264.7 macrophage cell line treated with or without LPS. Quantitative real-time PCR (qRT-PCR) analysis revealed that treatment with F-AgÅPs alone induced no profound effect on the expression of pro-inflammatory factors including interleukin-1*α* (*Il-1α*), *Il-1β*, *Il-6*, and tumor necrosis factor-*α* (*Tnf-α*), but caused a significant increase in the expression of anti-inflammatory factor *Il-10*, as compared with the vehicle-treated cells (**Figure 4F**). LPS treatment remarkably increased the expression of these pro-inflammatory factors in RAW264.7 cells, while the effect was profoundly suppressed by co-treatment with F-AgÅPs (**Figure 4F**). The expression of *Il-10* was also enhanced after exposure to LPS, whereas co-incubation with F-AgÅPs further increased the mRNA level of this anti-inflammatory factor, but only by trend (**Figure 4F**). The protein levels of a class of inflammatory cytokines released into the cultures of RAW264.7 cells were examined by the BD™ cytometric bead array (CBA) mouse inflammation kit combined with flow cytometry. As shown in **Figure 4G**, F-AgÅPs markedly decreased the levels of pro-inflammatory IL-6, TNF-α, and monocyte chemoattractant protein-1 (MCP-1) in the LPS-treated RAW264.7 cells, while the changes were not observed in the vehicle-treated cells. No notable differences were observed in the protein levels of IL-12p70 and interferon-γ (IFN-γ) between different groups (**Figure 4G**). These results suggest that F-AgÅPs have the ability to down-regulate the inflammatory responses in the LPS-activated macrophages, and enhance the anti-inflammatory activity of the quiescent macrophages.

### F-AgÅPs alleviate CLP-induced fatal sepsis

**Figure 5:**
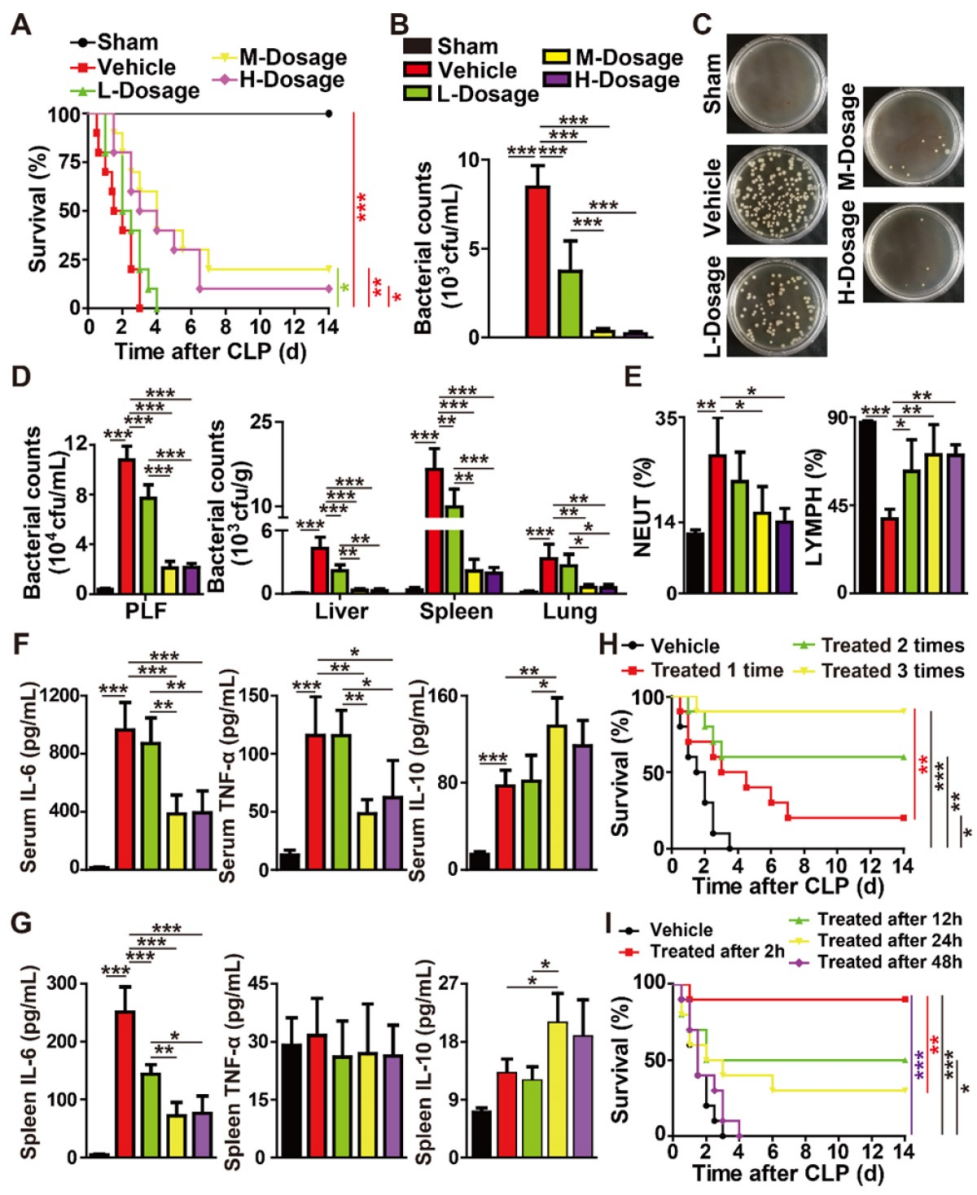
**F-AgÅPs alleviate CLP-induced fatal sepsis. (A)** Survival curves of the vehicle-treated sham mice and CLP mice treated with F-AgÅPs or vehicle one time at 2 h after surgery. H-Dosage: high dosage; M-Dosage: middle dosage; L-Dosage: low dosage. Vehicle indicates normal saline (solvent of F-AgÅPs). *n* = 10 *per* group. **(B-C)** Blood samples from sham or CLP mice receiving different treatments at 24 h after surgery were spread onto agar plates. Bacterial colony numbers were shown in (**B**) and the representative images of bacterial colonies were displayed in (**C**). *n* = 5 *per* group. **(D)** The numbers of bacterial colonies detected in PLF and homogenates of liver, spleen, and lung. *n* = 5 *per* group.** (E)** The percentages of neutrophils (NEUT) and lymphocytes (LYMPH) assessed by routine blood test. *n* = 5 *per* group. **(F-G)** Protein level analysis of IL-6, TNF-α, and IL-10 in blood (**G**) and spleen homogenates (**G**) by a CBA inflammation kit. *n* = 5 *per* group. **(H)** Survival curves of CLP mice treated with vehicle (normal saline) or middle dose of F-AgÅPs for one time (at 2 h after surgery), two times (at 2 h and 24 h after surgery) or three times (at 2 h, 24 h and 48 h after surgery). *n* = 10 *per* group. **(I)** Survival curves of CLP mice receiving three times injections of vehicle (normal saline) or middle dose of F-AgÅPs started at 2 h, 12 h, 24 h, or 48 h after surgery. *n* = 10 *per* group. ^*^P < 0.01, ^**^P < 0.01, ^***^P < 0.001.

We next studied whether the intravenous injection of different doses of F-AgÅPs for one time at 2 h after surgery could induce benefits in mouse models with CLP-induced high-grade sepsis (100% lethality within 4 days after CLP induction). Consistent with the result in previous study 23, the mice that were subjected to CLP and treated with normal saline (vehicle of F-AgÅPs) only all died within a very short time (≤ 3 days) (**Figure 5A**). A single injection of high (H-Dosage; 4.5 mg/kg) and especially middle (M-Dosage; 3.0 mg/kg) dosages of F-AgÅPs resulted in a significant increase of the survival rate in CLP mice, whereas low dosage (L-Dosage; 1.5 mg/kg) of F-AgÅPs only slightly extended the survival time of CLP mice (**Figure 5A**).

At 24 h after surgery, the blood samples of the sham-operated mice and CLP mice receiving a single injection of vehicle or F-AgÅPs were obtained for bacterial colony counting assay. As indicated by the quantification of bacterial colony numbers and the representative images of bacterial colonies in **Figure 5**, **B** and **C**, a large number of bacterial colonies were observed in blood samples from the vehicle-treated CLP mice, whereas no bacterial colonies were detected in blood samples from the vehicle-treated sham mice. The bacterial counts in blood were markedly decreased in the F-AgÅPs-treated CLP mice, especially in those treated with the middle or high doses of F-AgÅPs (**Figure 5**, **B** and **C**). Abundant bacteria were also detected in peritoneal lavage fluid (PLF) and homogenates of liver, lung, and especially spleen tissues of the vehicle-treated CLP mice at 24 h after surgery, but bacterial colonization in these sites were strikingly reduced after injection of F-AgÅPs, especially when given at the middle or high doses (**Figure 5D**).

**Figure S1A** shows that CLP did not affect the weights of liver, lung, heart, brain, and kidney in mice at 24 h after surgery, but induced a trend of increase in the weight of spleen, an important organ for the host-protective response against bacterial infection. However, the CLP-induced trend of increase in spleen weight was not observed in the mice treated with F-AgÅPs at the high and especially the middle dosage (**Figure S1A**). Hematoxylin and eosin (H&E) staining showed a much higher level of inflammatory cell accumulation in spleen tissues compared with other tissues in the vehicle-treated CLP mice (**Figure S1B**), consistent with the increase of spleen weight and the higher level of bacterial colonization in spleen tissues of these mice. Inflammatory cell infiltration was profoundly attenuated after treatment with F-AgÅPs and the reduction of inflammation was much notable in CLP mice treated with the middle or high doses of F-AgÅPs (**Figure S1B**).

Routine blood test revealed that CLP caused a significant increase in the percentage of neutrophils (NEUT%), a major factor contributing to uncontrolled inflammation, whereas the percentage of lymphocytes (LYMPH%) was markedly reduced after CLP (**Figure 5E** and **Table S1**). In CLP mice, however, F-AgÅPs at the middle or high doses remarkably reversed the opposite changes of these two parameters, while the alterations were milder in group receiving low dose of F-AgÅPs (**Figure 5E** and **Table S1**). F-AgÅPs treatment also significantly suppressed the CLP-induced up-regulation of monocytes (an important participator of inflammation; **Table S1**). Inflammatory factor analysis with a CBA mouse inflammation kit indicated that the vehicle-treated CLP mice exhibited marked increases in the levels of pro-inflammatory IL-6, TNF-α, and MCP-1, as well as the level of anti-inflammatory IL-10 in serum (**Figure 5F** and **Figure S1C**) or/and spleen homogenates (**Figure 5G** and **Figure S1D**). In the F-AgÅPs-treated CLP mice, the levels of these pro-inflammatory factors in serum or/and spleen homogenates were considerably reduced compared with the vehicle-treated CLP mice, particularly when F-AgÅPs were injected at the middle or high dosages (**Figure 5**, **F** and **G**; **Figure S1**, **C** and** D**). The serum and spleen concentration of anti-inflammatory IL-10, however, was further increased after treating F-AgÅPs at the middle or high dosages (**Figure 5**, **F** and **G**). There were no statistically significant differences in the levels of IL-12p70 and IFN-γ in serum and spleen homogenates between different groups (**Figure S1**, **C** and** D**).

Together, the above findings suggest that a single intravenous injection of F-AgÅPs can enhance the survival of mice with CLP-induced fatal sepsis, which is likely due to the reduction of bacterial burden and inflammation.

We next explored whether the multiple-dose intravenous administrations of F-AgÅPs could provide better protection against CLP-induced fatal sepsis. As F-AgÅPs at the middle dose was sufficient to induce potent benefits in CLP mice comparable to that achieved with high dose of F-AgÅPs, we chose the middle dose for further experiments. As indicated by the Kaplan-Meier survival curves, none (0/10) of the vehicle-treated CLP mice survived beyond day 4 after surgery, whereas 20% (2/10), 60% (6/10), and 90% (9/10) of the F-AgÅPs-treated CLP mice survived the entire 14-day observation period in one time (at 2 h after surgery), two times (at 2 h and 24 h after surgery), and three times (at 2 h, 24 h, and 48 h after surgery) treatment groups (**Figure 5H**), indicating that increasing the frequency of F-AgÅPs treatment leads to better outcomes. However, the pro-survival efficacy of the intravenous injection of F-AgÅPs for three times (given at 0 h, 24 h, and 48 h after the first administration) was only notable at 2 h, 12 h, or 24 h after the CLP surgery, and the survival rate was much higher when the treatment was started as early as possible (**Figure 5I**). Injection of F-AgÅPs started at 48 h after CLP failed to induce a significant long-term survival benefit in mice (**Figure 5I**), indicating that F-AgÅPs treatment should be started within 48 h of infection onset.

### F-AgÅPs protect against lethal* E. coli* bloodstream infection

**Figure 6:**
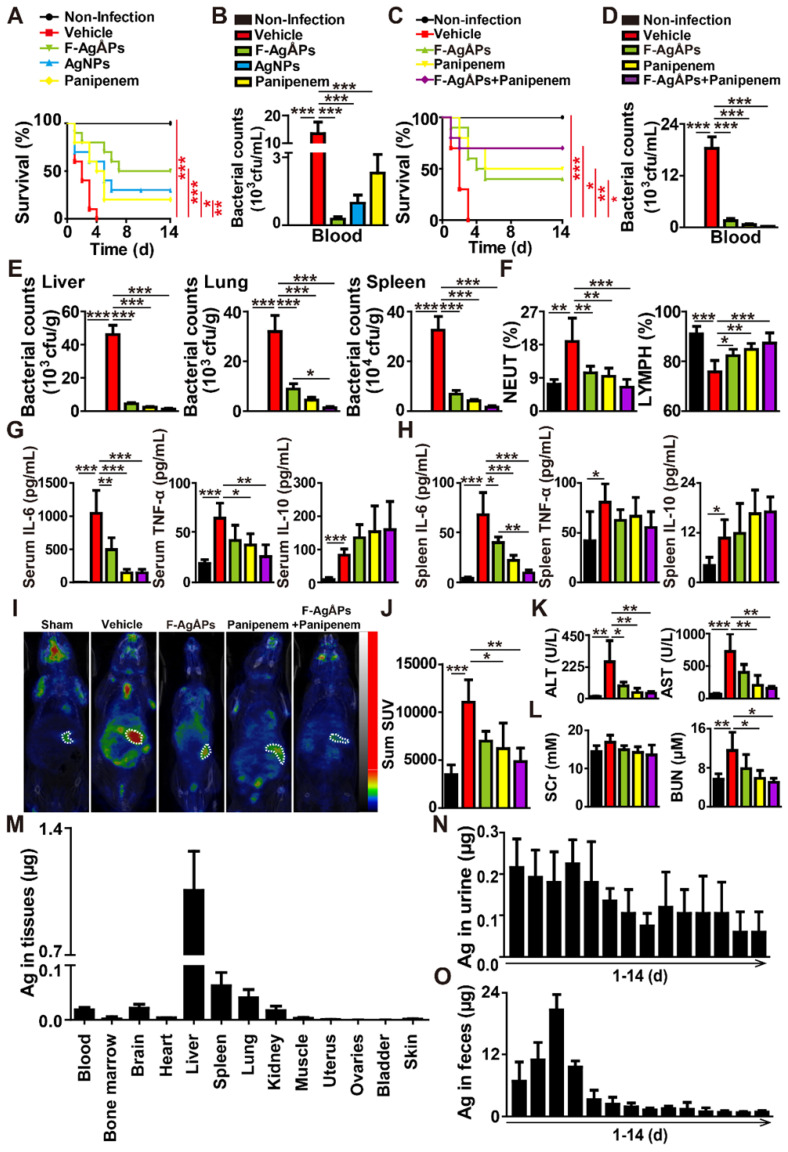
** F-AgÅPs protect against lethal* E. coli* bloodstream infection. (A)** Survival curves of the vehicle-treated non-infected mice and the carbapenem-resistant* E. coli*-infected mice receiving three times injections of vehicle, F-AgÅPs, AgNPs, or panipenem. Vehicle indicates normal saline (solvent of F-AgÅPs). *n* = 10 *per* group. **(B)** The numbers of bacterial colonies detected in blood samples from mice in (**A**) at 24 h after infection.* n* = 5 *per* group. **(C)** Survival curves of the vehicle-treated non-infected mice and the carbapenem-sensitive multi-drug resistant ESBL-producing *E. coli*-infected mice receiving three times injections of vehicle, F-AgÅPs, panipenem, or F-AgÅPs + panipenem. Vehicle indicates normal saline (solvent of F-AgÅPs). *n* = 10 *per* group. **(D)** The numbers of bacterial colonies detected in blood samples from mice in (**C**) at 24 h after infection.* n* = 5 *per* group. **(E)** The numbers of bacterial colonies detected in homogenates of liver, spleen, and lung tissues.* n* = 5 *per* group. **(F)** The values of NEUT% and LYMPH% tested by routine blood test. *n* = 6-7 *per* group. **(G-H)** Protein level analysis of IL-6, TNF-α, and IL-10 in blood (**G**) and spleen homogenates (**H**) by a CBA inflammation kit. *n* = 5 *per* group. **(I)** Representative PET/CT images of the vehicle (normal saline)-treated non-infected mice and the *E. coli*-infected mice in different groups at 20 min after injection of 18F-FDG. **(J)** Quantification of the standardized uptake value (SUV) for 18F-FDG in the areas of spleen tissues. *n* = 3-5 *per* group. **(K-L)** The serum levels of indicators revealing liver (ALT and AST; **K**) and kidney (SCr and BUN; **L**) injuries. *n* = 4-5 *per* group. **(M)** ICP-MS analysis of silver contents in blood, bone marrow, and major tissues from the F-AgÅPs-treated *E. coli*-infected mice at days 14 after infection. *n* = 4. **(N-O)** Daily excretion levels of silver in urine (**N**) and feces (**O**) during the 14-day observation period. *n* = 4-5. ^*^P < 0.01, ^**^P < 0.01, ^***^P < 0.001.

We then generated the mouse models of bloodstream infection using the clinically isolated carbapenem-resistant *E. coli* or the carbapenem-sensitive multi-drug-resistant ESBL-producing *E. coli* at the lethal dosage, and tested whether three times intravenous injections of F-AgÅPs (3.0 mg/kg) at 2 h, 24 h, and 48 h after infection could rescue the mice from different strains of *E. coli*-induced life-threatening condition. The protective effects of the similarly sized AgNPs and panipenem (a carbapenem antibiotic for treating multi-drug resistant ESBL-producing bacterial infection) were also evaluated in the carbapenem-resistant *E. coli*-induced blood infection. As evidenced by the Kaplan-Meier survival curves (**Figure 6A**), all the vehicle-treated infected mice died within 4 days of bacterial challenge. AgNPs and panipenem could induce a marked increase in the survival rate of the *E. coli*-infected mice, but the pro-survival effect was lower than that of F-AgÅPs (**Figure 6A**). Bacterial colony counting assay showed much lower bacterial loads in blood of the F-AgÅPs-, AgNPs-, or panipenem-treated *E. coli*-infected mice compared with the vehicle-treated group at 24 h after infection, whereas the ability of F-AgÅPs to reduce blood bacterial loads was higher than that of AgNPs and panipenem, but only by trend (**Figure 6B**). These results indicate that F-AgÅPs possess greater protective effects against the carbapenem-resistant *E.coli*-induced infection compared with panipenem and AgNPs with similar sizes.

In the carbapenem-sensitive multi-drug resistant *E. coli*-infected mice, we further assessed whether the combination of F-AgÅPs with panipenem could achieve better results. As shown in **Figure 6C**, both F-AgÅPs and panipenem were able to increase the survival of the carbapenem-sensitive multi-drug resistant *E. coli*-infected mice, but the pro-survival effect of panipenem was slightly higher than that of F-AgÅPs (**Figure 6C**). The combination of F-AgÅPs and panipenem induced a further increase in the survival rate of the *E. coli*-infected mice (**Figure 6C**). Bacterial colony counting assay showed that panipenem exhibited a trend of higher ability than F-AgÅPs to reduce bacterial loads in blood (**Figure 6D**) as well as in homogenates of liver, lung, and spleen tissues (**Figure 6E**), whereas co-treatment with F-AgÅPs and panipenem was slightly more or much more effective than F-AgÅPs or panipenem alone in reducing bacterial colonization (**Figure 6**, **D** and **E**).

F-AgÅPs also potently suppressed the carbapenem-sensitive multi-drug resistant* E. coli* infection-induced increases in the weight of spleen (**Figure S2A**), the accumulation of inflammatory cells in spleen tissue (**Figure S2B**), the value of NEUT% (**Figure 6F** and **Table S2**), and the levels of pro-inflammatory factors (including IL-6, TNF-α, and MCP-1) in serum (**Figure 6G** and **Figure S2C**) or/and spleen homogenates (**Figure 6H** and **Figure S2D**), but most of these effects were slightly lower than that of panipenem, as evidenced by weight measurement and H&E staining of the major organs, routine blood test, and inflammatory factor analysis based on a CBA immunoassay kit, respectively. The stimulatory effect of F-AgÅPs on the accumulation of anti-inflammatory IL-10 in serum and spleen was also mildly lower than that of panipenem (**Figure 6**, **G** and **H**). However, most of these changes in F-AgÅPs + panipenem co-treatment group were much more obvious than that of mice treated with F-AgÅPs or panipenem alone (**Figure 6F-H** and**Figure S2A-D**). 18F-fluorodeoxyglucose (18F-FDG) is a radiotracer that is used to measure the glycolytic activity and a higher level of 18F-FDG uptake is demonstrated in inflammatory lesions 24, 25. Whole body imaging with 18F-FDG-PET/CT further confirmed that the combination of F-AgÅPs and panipenem caused a greater inhibition of inflammation in the *E. coli*-infected mice, as indicated by the trend of lower level of 18F-FDG uptake in F-AgÅPs + panipenem group compared to F-AgÅPs or panipenem group (**Figure 6**, **I** and **J**).

Hepatic and renal function tests revealed that *E. coli* infection for 24 h resulted in significant increases in the levels of serum alanine transaminase (ALT), aspartate aminotransferase (AST), and blood urea nitrogen (BUN) (**Figure 6**, **K** and **L**; **Table S3**), indicating the impairment of liver and renal function. Treatment with F-AgÅPs, panipenem, or F-AgÅPs + panipenem for 24 h markedly suppressed the increases of these parameters, but these protective changes were slightly milder in F-AgÅPs group compared with panipenem and especially F-AgÅPs + panipenem groups (**Figure 6**, **K** and **L**; **Table S3**), suggesting the superiority of the combined use of F-AgÅPs and panipenem compared with their single use in the carbapenem-sensitive multi-drug resistant *E.coli*-induced infection.

ICP-MS analysis was conducted to assess the residual silver in blood, bone marrow, and major tissues of the F-AgÅPs-treated the carbapenem-sensitive multi-drug resistant *E.coli*-infected mice at the end of the 14-day observation period. As shown in **Figure 6M**, the liver, spleen, and lung tissues of mice exhibited higher rates of silver retention compared with other tissues, consistent with that observed in the tumor-bearing mice after F-AgÅPs injection in our previous studies 10, 11. Nevertheless, the total amount of silver detected in these tissues was much lower than the total dose of F-AgÅPs given, only accounting for 2.59% (**Figure 6M**), suggesting that a large amount of silver might has been eliminated from the mouse body. **Figure 6**, **N** and **O** show that a much higher level of silver was detected in feces, but not in urine. After three times injections at 2 h, 24 h, and 48 h after infection, the total amount of the excreted silver *via* urine and feces accounted for 2.36% and 79.18% of the total body burden of F-AgÅPs (**Figure 6**, **N** and **O**), which further confirmed the successful elimination of most of the silver after F-AgÅPs administration.

## Discussion

Effective and timely anti-bacterial therapy is critical for improving survival of patients with severe bacteremia and sepsis 26. The increase of antibiotic-resistant bacteria makes it difficult to choose adequate anti-bacterial treatment 26.

Owing to the anti-bacterial properties, AgNPs-based medical and consumer products such as burn ointments, wound dressings, medical device coatings, and protective clothing have been widely used in healthcare 9, 27. The current approaches for delivering AgNPs for therapeutic uses mainly depend on skin contact or implantation as coatings 28. Exposure to AgNPs in humans can also occur accidentally through the inhalation or oral routes 28. Intravenous route is often used for drug administration due to its rapid delivery, immediate drug effect, and high bioavailability 11, 29. However, the use of this route for AgNPs treatment is primarily present in animal studies for testing the pharmacokinetics, tissue distribution, excretion, and toxicity of AgNPs 10, 30-37. For therapeutic uses in systemic infection, there is a study by Sun *et al*. showing the anti-bacterial and anti-inflammatory efficacy of the combined use of AgNPs with small interfering RNA (siRNA) and anti-oxidant/anti-bacterial quercetin by intravenous route in nude mice with *Bacillus subtilis*-induced blood infection 38. The currently used AgNPs are mainly synthesized by chemical routes and often stabilized by citrate, starch, polyvinylpyrrolidone, amphiphilic polymer, albumin, *etc*. 10, 30-34, 37, 39-41. However, in our previous 11, 12 and present studies, we used a pure physical method with our self-developed automatic evaporation-condensation system that could efficiently fabricate ultrasmall AgÅPs without using any hazardous or toxic chemical reducing agents, thus avoiding unwanted side effects if they were intravenously administered to animals. We utilized fructose, which has previously been used as a surfactant/stabilizer to prevent uncontrolled particle growth, agglomeration, and precipitation of the Ag nanostructures 42-45, to disperse and stabilize AgÅPs. Our evidences demonstrated that fructose could be successfully coated on AgÅPs and made F-AgÅPs well dispersed and highly stable in aqueous solution. Since fructose is one of the major carbon sources for most organisms, the use of fructose as a stabilizer/dispersant may enhance the biocompatibility of the silver particles. Expectedly, we found that F-AgÅPs had a good biocompatibility with human or mouse cells. F-AgÅPs at the bacteria-sensitive dosage did not only show no obvious toxicity on healthy cells, but also effectively protected these cells from bacteria or bacterial toxins-induced cell injuries. These features make F-AgÅPs very suitable for as an intravenously applied anti-bacterial agent.

In the present study, we provided the first evidence that the intravenous delivery of F-AgÅPs within 48 h effectively rescued mice from lethal bacteremia and sepsis induced by CLP surgery or* E. coli* infection. In the carbapenem-resistant *E.coli*-infected mice, the pro-survival and anti-bacterial efficacy of F-AgÅPs was higher than the commercial similarly sized AgNPs and carbapenem antibiotic panipenem, one of the best available antibiotics against multi-drug resistant bacteria 46. In the carbapenem-sensitive multi-drug resistant *E. coli*-infected mice, the protective effects of F-AgÅPs were just slightly lower than panipenem. When the mice were co-treated with F-AgÅPs and panipenem, better improvements were observed. Future studies are worthwhile to determine whether there exists a synergistic effect in the combination of F-AgÅPs and panipenem. After three injections within 48 h, most of the injected F-AgÅPs were eliminated from the mouse body through feces at two weeks after infection. These findings suggest the potential of F-AgÅPs as a safe and efficient intravenous agent for treating serious bacterial infections.

Besides the slightly smaller sizes, we hypothesized that at least two other reasons may account for the different effectiveness of our self-prepared F-AgÅPs and the commercial similarly sized AgNPs in inducing bacterial damage and protecting against bacterial infection-induced host injury. Firstly, since fructose can be transported and utilized by many bacteria 14-19, F-AgÅPs may interact with the bacteria more easily and efficiently than AgNPs. Then, F-AgÅPs enter into the bacteria and induce more severe bacterial damage. Owing to the small size, F-AgÅPs may also directly penetrate bacterial cell wall to cause intracellular injuries after interaction with the recipient bacteria. Secondly, with the utilization of fructose by the recipient bacteria, AgÅPs can released from fructose. Since these silver particles exhibit much smaller sizes than AgNPs, they will cause more serious and fatal injuries in bacterial membrane and subcellular compartments due to relative larger surface areas. A limitation of our study is that we did not determine the surface areas of F-AgÅPs, all AgÅPs within F-AgÅPs, and AgNPs showing similar sizes with F-AgÅPs. More in-depth studies are required to elucidate the detailed mechanisms by which F-AgÅPs induce much greater anti-bacterial and host-protective effects than AgNPs with similar sizes.

Bacteria have developed multiple strategies to invade their host, evade, or overcome the host defenses, and reach blood or distant organs where they survive, proliferate, and cause severe cellular injuries and organ dysfunction 47, 48. Bacteria can employ different virulence factors, such as the secreted toxins, to protect them from the host immune system and establish a replicative niche within the host 47. *S. aureus* is a major cause of sepsis and α-hemolysin is an archetypal pore-forming toxin that plays a critical role in the pathogenesis of *S. aureus* infection by inducing the lysis of many types of cells, such as RBCs, lymphocytes, macrophages, and alveolar epithelial cells 49. *S. aureus* mutant strains lacking α-hemolysin exhibit loss of virulence and fail to cause death in mice with lung infection 50. Previous evidence has shown that AgNPs can interact with the membrane and intracellular proteins and disturb the metabolic processes in bacteria, which eventually lead to bacterial death 51. In this study, we found that F-AgÅPs could not only directly kill different *S. aureus* strains, but also have the ability to sequester α-hemolysin to abrogate its cytotoxic activity on hepatocytes and endothelial cells, which would prevent host tissue damage and finally facilitate the further elimination of the bacteria by the host immune system. Our results suggest the bacterial-killing and host-protecting dual functions of F-AgÅPs.

Sepsis is a highly lethal condition characterized by systemic inflammation, resulting from uncontrolled host immune responses to a serious infection 5. The strong activation of whole-body inflammation is essential for preventing against the invading pathogens, but may cause detrimental consequences such as multiple-organ failure 52, 53. As the major members of the innate immune system, neutrophils, and macrophages recognize the microbial components such as the bacterial LPS and participate in the clearance of microbes during sepsis 54. When persistently activated, these cells can cause damage to nearby healthy cells and tissues by continuous production of pro-inflammatory factors and other toxic substances 54. Lymphocytes, however, exhibit a significant decline due to apoptosis during bacterial infection, which results in an immunosuppressive state in septic patients and makes the patients vulnerable to new infections 54, 55. The CLP model is a frequently used model for polymicrobial sepsis, in which sepsis originates from an abdominal septic focus, followed by the translocation of bacteria and their toxins into the circulation and subsequent the induction of systemic inflammation 23. In our study, the mice subjected to CLP surgery or intravenous infusion of live *E. coli* showed a prominent increase of neutrophils and a significant decrease of lymphocytes, but the alterations were remarkably reversed by F-AgÅPs. The CLP- or *E. coli* infection-induced increase of inflammation in blood and spleen as well as the up-regulation of indicators revealing liver and kidney injuries were also markedly suppressed in mice intravenously injected with F-AgÅPs. The bactericidal and toxin-binding action of F-AgÅPs may contribute importantly to their inhibition on neutrophil accumulation, lymphocyte reduction, inflammatory cell infiltration, pro-inflammatory cytokine overproduction, and organ dysfunction, thus rescuing the mice from fatal sepsis. However, it should be noted that F-AgÅPs could suppress the production of pro-inflammatory factors and enhance the anti-inflammatory cytokine IL-10 in the *E. coli* LPS-activated macrophages *in vitro*, suggesting that the direct inhibition of macrophage inflammation may be another critical factor contributing to the protective effects of F-AgÅPs against sepsis. The detailed mechanism by which F-AgÅPs down-regulate macrophage inflammation still requires future investigation.

## Materials and Methods

Approval for this study was obtained from the Ethics Committee of Xiangya Hospital of Central South University. Animal care and experiments were conducted following the guidelines of the Department of Laboratory Animals of Central South University.

### Preparation of AgÅPs and F-AgÅPs

Since silver particles with smaller sizes have stronger anti-bacterial potential 13 and fructose is often used to stabilize and disperse silver particles 42-45, we prepared silver particles reaching Ångstrom-scale and coated fructose on these ultrasmall particles (AgÅPs) for assessing the anti-bacterial activities of F-AgÅPs. The procedures for the preparation of AgÅPs using our self-developed automatic evaporation-condensation system were detailed in our published studies 11, 12, 56. Briefly, the air in the system was removed using the vacuum pump, followed by filling the system with the protective gas Argon (100 to 1000 kPa). The pure silver wires with diameters of 0.1 to 0.8 mm and lengths of 8 to 12 cm were loaded into the silver wire supplier and continuously fed to the explosion chamber. When the pluse power supply was turned on and the silver wires contacted with the electrode plate in the chamber, 25 to 45 kV high voltages with arc lightning were generated and the silver wires could be exploded and gasified to produce silver vapor. After flowing in the buffer tank with the shielding gas Argon under the action of the electric blower, the silver vapor became more uniformly distributed and then entered into the rapid cooling system, where the silver vapor was coagulated to generate silver particles. After being dispersed by a high intensity ultrasonic dispersing device at 15 kW and 15 kHz, the silver particles were demagnetized (voltage: 24 to 36 V; current: 1000 mA; frequency: 23 Hz) to prevent aggregation. Then, the particles were transferred into the particle collection system and size-graded by three series-connected collectors under 1.2 m s^-1^ gas flow rate. Silver particles with three size ranges (largest: > 20 nm; medium-sized: 50 Å < diameters < 20 nm; smallest: 1 to 50 Å) were obtained in different collectors due to their different sedimentation velocities and the smallest silver particles (AgÅPs) were used in this study. The naked AgÅPs were then coated with fructose to obtain F-AgÅPs using our recently reported optimized method 12. In brief, AgÅPs were mixed with fructose to reach a final concentration of 0.5 g L^-1^ and 1.0 g L^-1^, respectively. After being dispersed for 15 s by an ultrasonic device at 75 kHz and 3 kW, the mixture was stood for 0.5 h and then subjected to ultrasonic atomization at 15 kHz and 12 kW, followed by collection of the atomized F-AgÅPs solution with a 60 square meter condenser.

### Characterization of AgÅPs and F-AgÅPs

The morphologies and diameters of AgÅPs and F-AgÅPs were detected with a HT770 TEM (Hitachi, Tokyo, Japan). XRD analysis was carried out to assess the crystal structures of AgÅPs and F-AgÅPs using a X'Pert Pro MPD diffractometer (PANalytical, Holland). EDS analysis was conducted to test the chemical composition of AgÅPs and F-AgÅPs. UV-Vis-NIR and FT-IR spectra of AgÅPs and F-AgÅPs were recorded on a PerkinElmer Lambda750 spectrophotometer (Waltham, Massachusetts, USA) and a Thermo Scientific Nicolet iS5 spectrometer (Madison, WI, USA), respectively. DLS analysis was performed to assess the zeta potential of F-AgÅPs in deionized water using a Zetasizer Nano ZS analyzer (Malvern Instruments Ltd, UK). To evaluate the dispersion and stability in aqueous solution, AgÅPs and F-AgÅPs were dissolved in deionized water and left for 30 days at room temperature. The suspensions of AgÅPs and F-AgÅPs were photographed and silver contents in the supernatant were measured by ICP-MS.

### Bacterial strains

*S. aureus* ATCC25923, *S. aureus* ATCC29213, *E. coli* ATCC25922, *P. aeruginosa* ATCC27853, *S. pneumoniae* ATCC27336,* S. pyogenes* ATCC19615, and the clinically isolated multi-drug-resistant *S. aureus* strain, carbapenem-sensitive multi-drug-resistant ESBL-producing *E. coli* strain, and carbapenem-resistant *E. coli* strain were provided by the Department of Clinical Laboratory in Xiangya Hospital of Central South University.

### Cell culture

Bacteria were incubated in nutrient broth at 37 °C with shaking at 300 rpm before the downstream assays. The culture medium of the clinically isolated multi-drug-resistant *S. aureus* was obtained and centrifuged at 2800 × g for 10 min. The culture supernatant was harvested and stored at -80 °C before use. HMECs were grown in MCDB131 medium (Gibco, Grand Island, NY, USA) with 10% fetal bovine serum (FBS; Gibco) and 1% GlutaMAX (Gibco). VSMCs were maintained in F12K medium (Hyclone, Logan, UT, USA) containing 10% FBS. LO2 cells were incubated in RPMI 1640 medium (Biological Industries, Beit Haemek, Israel) with 10% FBS. bEnd.3 and RAW264.7 cells were cultured in high-glucose DMEM (Gibco) with 10% FBS. Cells were grown at 37 °C with 5% CO_2_.

### Agar disk diffusion assay

The filter paper disks (6 mm diameter) were infiltrated with undiluted F-AgÅPs (500 ng μL^-1^) or vehicle for 12 h and then were placed onto Muller-Hinton (MH) agar plates that were inoculated with the suspension of the tested bacteria (1 × 10^8^ CFU mL^-1^; 150 μL *per* dish). After incubation for 12 h at 37 °C, digital photos of the agar plates were obtained and the diameters (mm) of the bacterial growth inhibition zones were assessed.

### Determination of MIC and MBC

Two-fold serial dilutions of F-AgÅPs (80, 40, 20, 10, 5, and 2.5 ng μL^-1^) were prepared in MH broth and added to 96-well culture plates (100 μL *per* well). The tested bacteria (1 × 10^8^ CFU mL^-1^; 5 μL *per* well) were added to F-AgÅPs solution and cultured at 37 °C with shaking at 300 rpm for 18 h. The bacteria cultured in MH broth without F-AgÅPs served as positive controls and the blank control was MH broth without bacteria. Bacterial growth was evaluated by a microplate reader (Thermo Fisher Scientific) at 595 nm. The MIC of F-AgÅPs against the tested bacteria was recorded as the lowest concentration at which no visible bacterial growth was detected. To determine MBC, 5 μL of the suspension in each well was diluted to 100 μL with MH broth and spread onto the LB agar plate. The MBC of F-AgÅPs was defined as the lowest concentration at which no bacterial colonies were observed after 24 h of incubation at 37 °C.

### Bacterial colony counting assay

To compare the effect of F-AgÅPs and AgNPs (ranged from 10 to 15 nm and stabilized by sodium citrate; Aladdin, Shanghai, China) on bacterial colony formation, F-AgÅPs at the concentrations of MIC values against the tested bacteria, AgNPs at the same concentrations, or an equal volume of vehicle (normal saline) was mixed with the bacterial solution (1 × 10^6^ CFU mL^-1^) and incubated at 37 °C for 8 h. After being diluted with LB broth for 10000 folds, 100 μL of the bacterial solution was spread on the LB agar plate. After 12 h of incubation, bacterial colonies were photographed, counted, and expressed as CFU *per* milliliter of bacterial solution. To explore the potential of F-AgÅPs as an anti-bacterial agent against blood infection *in vitro*, the tested bacteria were cultured in mouse blood and treated with F-AgÅPs or vehicle for 3 h 37 °C. The blood samples were then diluted with LB broth and processed for bacterial colony counting assay using procedures as above.

### Alamar blue assay

The tested bacteria (8 × 10^4^ CFU *per* well) were incubated in 96-well culture plates and treated with MIC doses of F-AgÅPs, AgNPs at the same concentrations, or vehicle (normal saline) in LB medium with alamar blue dye (10 μL *per* well; Yeasen). Mitomycin-C (5 µg/mL; Sigma‑Aldrich, St. Louis, MO, USA) was added to the cultures of different groups throughout the alamar blue assay to exclude the impact of bacterial proliferation. After incubation at 37 °C for 3 h, the resulting fluorescence was detected with a fluorescence microplate reader (Thermo Fisher Scientific) using excitation at 545 nm and emission at 590 nm. Bacterial survival (%) was represented by the ratio of the fluorescence intensity of the F-AgÅPs-treated cells to that of the control cells.

### Morphological and chemical analyses

The tested bacteria (1 × 10^8^ CFU mL^-1^) were treated with MIC doses of F-AgÅPs, AgNPs at the same concentrations, or vehicle (normal saline) for 3 h and then centrifuged at 2800 rpm for 5 min. After removing the supernatant, the bacterial pellets were fixed for 2 h with 2.5% glutaraldehyde and processed for TEM observation. The areas of interest in TEM sections were further subjected to *in situ* chemical composition analysis using EDS.

### Pretreatment of α-hemolysin and LPS with F-AgÅPs

To determine whether F-AgÅPs could bind to *S. aureus* α-hemolysin, 450 ng mL^-1^ α-hemolysin (Abcam, Cambridge, Britain) or 100 ng mL^-1^ *E. coli* LPS (Sigma‑Aldrich) was added to the culture of different cell types with or without F-AgÅPs (2.5 ng μL^-1^), to F-AgÅPs without cells, or to vehicle (PBS + normal saline) only. After incubation at 37 °C for 1 h, the samples were centrifuged at low-speed (400 × g) for 10 min to obtain the cell pellet. The culture supernatant was then subjected to high-speed centrifugation at 100, 000 × g for 2 h to obtain the F-AgÅPs pellet and soluble fraction. ELISA was conducted to assess the contents of α-hemolysin or LPS in the resulting low-speed pellet, high-speed pellet, and high-speed soluble fraction using two commercial kits from Jingmei Biotechnology (Dafeng, Jiangsu, China). To assess whether binding with F-AgÅPs could affect the cytotoxic activities of α-hemolysin, 2700 ng mL^-1^ α-hemolysin was pretreated with 15 ng μL^-1^ F-AgÅPs for 30 min, followed by high-speed centrifugation to obtain the pellet (F-AgÅPs binding with α-hemolysin) and the α-hemolysin-reduced supernatant. The vehicle (normal saline)-pretreated α-hemolysin (2700 ng mL^-1^) was also subjected to high-speed centrifugation and α-hemolysin in the supernatant served as the control group. Then, the vehicle- or F-AgÅPs-pretreated α-hemolysin at different concentrations (for LO2: 300 ng mL^-1^; for bEnd.3: 1200 ng mL^-1^; for RBC: 450 ng mL^-1^) was used for live/dead cell staining, CCK-8 assay, and hemolysis assay.

### Live/dead cell staining

To compare the effect of F-AgÅPs and AgNPs on survival of bacteria, the clinically isolated* S. aureus* and *E. coli* (1 × 10^8^ CFU mL^-1^) were treated with MIC doses of F-AgÅPs, AgNPs at the same concentrations, or an equal volume of vehicle (normal saline) for 3 h. To test whether F-AgÅPs could protect the human or mouse cells from bacteria or bacterial toxins-induced cell death, HMECs, bEnd.3, LO2, RAW264.7, and VSMCs (1 × 10^4^ cells mL^-1^) were treated with bacteria (1 × 10^6^ CFU mL^-1^), F-AgÅPs (for HMECs: 5 ng μL^-1^; for LO2, RAW264.7, and VSMCs: 10 ng μL^-1^; for bEnd.3: 15 ng μL^-1^), bacteria + F-AgÅPs, bacteria-derived culture supernatant, bacteria-derived culture supernatant + F-AgÅPs, F-AgÅPs-pretreated bacteria-derived culture supernatant, α-hemolysin (for LO2: 300 ng mL^-1^; for bEnd.3: 1200 ng mL^-1^), α-hemolysin + F-AgÅPs, vehicle- or F-AgÅPs-pretreated α-hemolysin (for LO2: 300 ng mL^-1^; for bEnd.3: 1200 ng mL^-1^), α-hemolysin-reduced supernatant, LPS (100 ng mL^-1^), LPS + F-AgÅPs, or an equal volume of vehicle (normal saline, normal saline + un-cultured medium (for culturing *S. aureus*), or PBS + normal saline) for 5 h. The treated cells were washed twice with assay buffer and stained with calcein-AM (4 μM) and PI solution (9 μM) using the reagents purchased from Yeasen Biotech (Shanghai, China). After incubation for 30 min at 37 °C, the cells were washed and photographed using a fluorescence microscope (Zeiss, Jena, Germany). The percentages of live cells (calcein-AM^+^PI^-^) were calculated.

### CCK-8 assay

CCK-8 analysis was conducted to test cell survival/growth as described previously 57, 58. Briefly, LO2, bEnd.3, HMECs, and RAW264.7 cells (5 × 10^3^ cells/well) were incubated in 96-well culture plates and treated with α-hemolysin (for LO2: 300 ng mL^-1^; for bEnd.3: 1200 ng mL^-1^), F-AgÅPs (for HMECs: 5 ng μL^-1^; for LO2, RAW264.7, and VSMCs: 10 ng μL^-1^; for bEnd.3: 15 ng μL^-1^), α-hemolysin + F-AgÅPs, LPS (100 ng mL^-1^), LPS + F-AgÅPs, vehicle- or F-AgÅPs-pretreated α-hemolysin (for LO2: 300 ng mL^-1^; for bEnd.3: 1200 ng mL^-1^), α-hemolysin-reduced supernatant, or an equal volume of vehicle (normal saline or PBS + normal saline) for 24 h, followed by incubation for another 3 h in fresh medium containing CCK-8 reagent (10 μL *per* well; 7Sea Biotech, Shanghai, China). Four wells only added with the fresh medium containing CCK-8 reagent served as the blank controls. The optical density (OD) values were tested at 450 nm by a microplate reader.

### Hemolysis assay

Rabbit whole blood was harvested in heparinized tubes, followed by centrifugation at 700 × g for 10 min to obtain RBC. The RBC pellet was washed with normal saline and diluted to 5% concentration by volume. The RBC suspension was treated with α-hemolysin (450 ng mL^-1^), F-AgÅPs (2.5 ng μL^-1^), α-hemolysin + F-AgÅPs, vehicle (normal saline)- or F-AgÅPs-pretreated α-hemolysin (450 ng mL^-1^), an equal volume of α-hemolysin-reduced supernatant, or an equal volume of vehicle (normal saline or PBS + normal saline). RBC treated with water served as positive control and the negative control was RBC treated with PBS. After 3 h of incubation at 37 °C with gentle shaking, the suspension was photographed and then centrifuged at 700 × g for 10 min. The absorbance of free hemoglobin in the supernatant was tested by a microplate reader at 541 nm. Hemolytic rate (%) = (absorbance of treated cells - absorbance of negative control cells) / (absorbance of positive control cells - absorbance of negative control cells) × 100.

### qRT-PCR

RAW264.7 macrophages were treated with F-AgÅPs (2.5 ng μL^-1^), LPS (100 ng mL^-1^), LPS + F-AgÅPs, or vehicle (PBS + normal saline) for 24 h. Total RNA from the treated cells was extracted and transcribed to cDNA, followed by qRT-PCR as described previously 59. The primer sequences for qRT-PCR were shown in **Table S4**.

### Animals and treatments

To investigate the therapeutic benefits of F-AgÅPs in CLP-induced fatal sepsis, 3-month-old female C57BL/6 mice were subjected to CLP surgery to induce high-grade sepsis, or just underwent a sham operation. The procedures were conducted as described previously 23. F-AgÅPs or vehicle (normal saline) was injected intravenously into CLP mice at the indicated dosages (4.5, 3.5 or 1.5 mg/kg), frequencies of administration (one, two, or three times) and starting time of treatment (at 2 h, 12 h, 24 h or 48 h after CLP surgery). The sham-operated mice were injected with the same volume of vehicle (normal saline) at the same timepoints. To explore whether F-AgÅPs could protect against *E. coli*-induced bloodstream infection, 3-month-old female C57BL/6 mice were administered intravenously with the clinically isolated carbapenem-resistant *E. coli* or the carbapenem-sensitive multi-drug-resistant ESBL-producing *E. coli* at the lethal dosage (1 × 10^9^ CFU bacteria in 100 μL normal saline). Control (non-infected) mice received normal saline only. At 2 h, 24 h, and 48 h after infection, the *E. coli*-infected mice were injected intravenously with F-AgÅPs (3.0 mg/kg in 100 μL normal saline), AgNPs (3.0 mg/kg), panipenem (10 mg/kg; D&C Chemicals, Shanghai, China), F-AgÅPs + panipenem, or vehicle (normal saline). The non-infected mice were treated with an equal volume of normal saline. Mouse survival (ten mice in each group) was determined over a 14-day observation period. For the remaining mice, the whole blood, PLF, and major organs (including liver, spleen, lung, heart, brain, and kidney) were obtained at 24 h after CLP surgery or *E. coli* infection and processed for downstream analyses.

### Assessment of bacterial load in blood and tissues

Samples of PLF were diluted with PBS by 200 folds; Samples of whole blood, homogenates of liver, spleen, and lung tissues were diluted with PBS by 50 folds. 100 μL of each diluted sample was spread on the LB agar plate and incubated at 37 °C for 12 h to count bacterial colonies. The numbers of CFU *per* milliliter of blood/PLF or *per* gram of tissue were calculated.

### Histological analysis

The liver, spleen, lung, heart, brain, and kidney tissues were weighed and fixed for 24 h using 4% paraformaldehyde. After dehydration with graded ethanol, the tissues were paraffin-embedded, cut into 4-μm-thick sections, and subjected to H&E staining with a kit (Servicebio, Wuhan, China) to assess histological changes. Images of the sections were obtained with an optical microscope (Olympus CX31, Tokyo, Japan).

### Routine blood test and hepatic/renal function tests

To assess the changes of hematologic indexes, whole blood samples were collected in EDTA-K2 containing test tubes for blood routine examination. Serum was harvested from the whole blood by centrifugation at 1000 ×g for 15 min, in order to measure the levels of hepatic and renal function indicators. These assessments were performed on automated instruments in Department of Clinical Laboratory in Xiangya Hospital.

### CBA assay

The levels of IL-6, IL-12p70, TNF-α, MCP-1, IFN-γ, and IL-10 in mouse serum and spleen homogenates were analyzed by a CBA mouse inflammation kit from BD Biosciences (San Jose, CA, USA) according to the manufacturer's protocol. To assess the effect of F-AgÅPs on macrophage inflammation *in vitro*, RAW264.7 cells were incubated with F-AgÅPs (2.5 ng μL^-1^), LPS (100 ng mL^-1^), LPS + F-AgÅPs, or vehicle (PBS + normal saline) for 2 days. The culture medium was obtained and centrifuged at 2000 × g for 10 min to remove dead cells and debris. The supernatant was collected for inflammatory factor analysis using the CBA mouse inflammation kit.

### 18F-FDG-PET/CT imaging

The non-infected or *E. coli*-infected mice were given different treatments for 24 h and fasted for 12 h during the second half of the 24 h monitoring period. 18F-FDG (150 μCi for each mouse) was injected into the mice by intravenous route and the mice were subjected to whole body imaging using a PET/CT scanner (nanoScan PET; Mediso), with a 10 cm field of view, a voltage of 35 kV, a current of 980 μA, and 110 μm slice thickness. The Nucline nanoScan was used to reconstruct the images of the mice and quantify the standardized uptake value (SUV) in the region of interest.

### Retention of silver in biofluids and tissues

At the end of the 14-day observation period, the whole blood, bone marrow, liver, spleen, lung, kidney, brain, heart, muscle, uterus, ovary, bladder, and skin tissues of the survived F-AgÅPs-treated *E. coli*-infected mice were obtained and cut into small pieces. After digestion in aqua regia, the samples were filtered and the contents of silver in the supernatant were analyzed by ICP-MS.

### Excretion of F-AgÅPs

After receiving three times injections of F-AgÅPs at 2 h, 24 h, and 48 h after infection, the *E. coli*-infected mice were monitored for up to 14 days. 24 h-urine and -feces of the survived F-AgÅPs-treated *E. coli*-infected mice were collected, weighed, and processed for silver measurement by ICP-MS.

### Statistical analysis

Two-group and multiple-group comparisons, respectively, were conducted using student's *t*-test (unpaired, two tailed) and one-way ANOVA followed by Bonferroni *post hoc* test with GraphPad Prism 8 software. Comparison of survival rates between groups was performed using Log-rank test. Differences were considered significant at *P* < 0.05.

## Supplementary Material

Supplementary figures and tables.

## Abbreviations

*S. aureus*: *Staphylococcus aureus*
*E. coli*: *Escherichia coli*
LPS: lipopolysaccharide
NPs: nanoparticles
AgNPs: silver nanoparticles
AgÅPs: Ångstrom-scale silver particles
F-AgÅPs: fructose-coated AgÅPs
PDK: pyruvate dehydrogenase kinase
CLP: cecal ligation and puncture
TEM: transmission electron microscope
XRD: X-ray diffraction
EDS: energy dispersive spectroscopy
UV-Vis-NIR: ultraviolet-visible near-infrared
FT-IR: fourier-transform infrared
DLS: dynamic light scattering
ICP-MS: inductively coupled plasma mass spectrometry
*P. aeruginosa*: *Pseudomonas aeruginosa*
*S. pyogenes*: *Streptococcus pyogenes*
ESBL: extended-spectrum beta-lactamase
MIC: minimum inhibitory concentration
MBC: minimum bactericidal concentration
LB: Luria-Bertani
PI: propidium iodide
HMECs: human microvascular endothelial cells
VSMCs: vascular smooth muscle cells
CM: culture supernatant
CCK-8: cell counting kit-8
RBC: red blood cell
ELISA: enzyme-linked immunosorbent assay
qRT-PCR: quantitative real-time PCR
IL-1α: interleukin-1α
IL-1β: interleukin-1β
IL-6: interleukin-6
IL-10: interleukin-10
IL-12p70: interleukin-12p70
TNF-α: tumor necrosis factor-α
CBA: cytometric bead array
MCP-1: monocyte chemoattractant protein-1
IFN-γ: interferon-γ
H&E: hematoxylin and eosin
PLF: peritoneal lavage fluid
NEUT: neutrophils
LYMPH: lymphocytes
18F-FDG: 18F-fluorodeoxyglucose
ALT: alanine transaminase
AST: aspartate aminotransferase
BUN: blood urea nitrogen
SCr: serum creatinine
FBS: fetal bovine serum
MH: Muller-Hinton
OD: optical density
PBS: phosphate buffer solution
SUV: standardized uptake value
ANOVA: analysis of variance
