# Supplementary material for: Fructose-coated Ångstrom silver prevents sepsis by killing bacteria and attenuating bacterial toxin-induced injuries
Source: Theranostics. 2021 Jul 13;11(17):8152–71. doi: 10.7150/thno.55334 (PMC8344005; doi:10.7150/thno.55334)
Supplement: Supplementary file 1 — Supplementary figures and tables. [file thnov11p8152s1.pdf]

Supporting Information

Fructose-coated Ångstrom silver prevents sepsis by killing bacteria and attenuating bacterial toxins-induced injuries

Hao Yin, Mao Zhou, Xia Chen, Teng-Fei Wan, Ling Jin, Shan-Shan Rao, Yi-Juan Tan, Ran Duan, Yu Zhang, Zhen-Xing Wang, Yi-Yi Wang, Ze-Hui He, Ming-Jie Luo, Xiong-Ke Hu, Yang Wang, Wei-Yi Situ, Si-Yuan Tang, Wen-En Liu, Chun-Yuan Chen*, Hui Xie*

* Corresponding authors: Hui Xie (huixie@csu.edu.cn); Chun-Yuan Chen (chency19@csu.edu.cn).

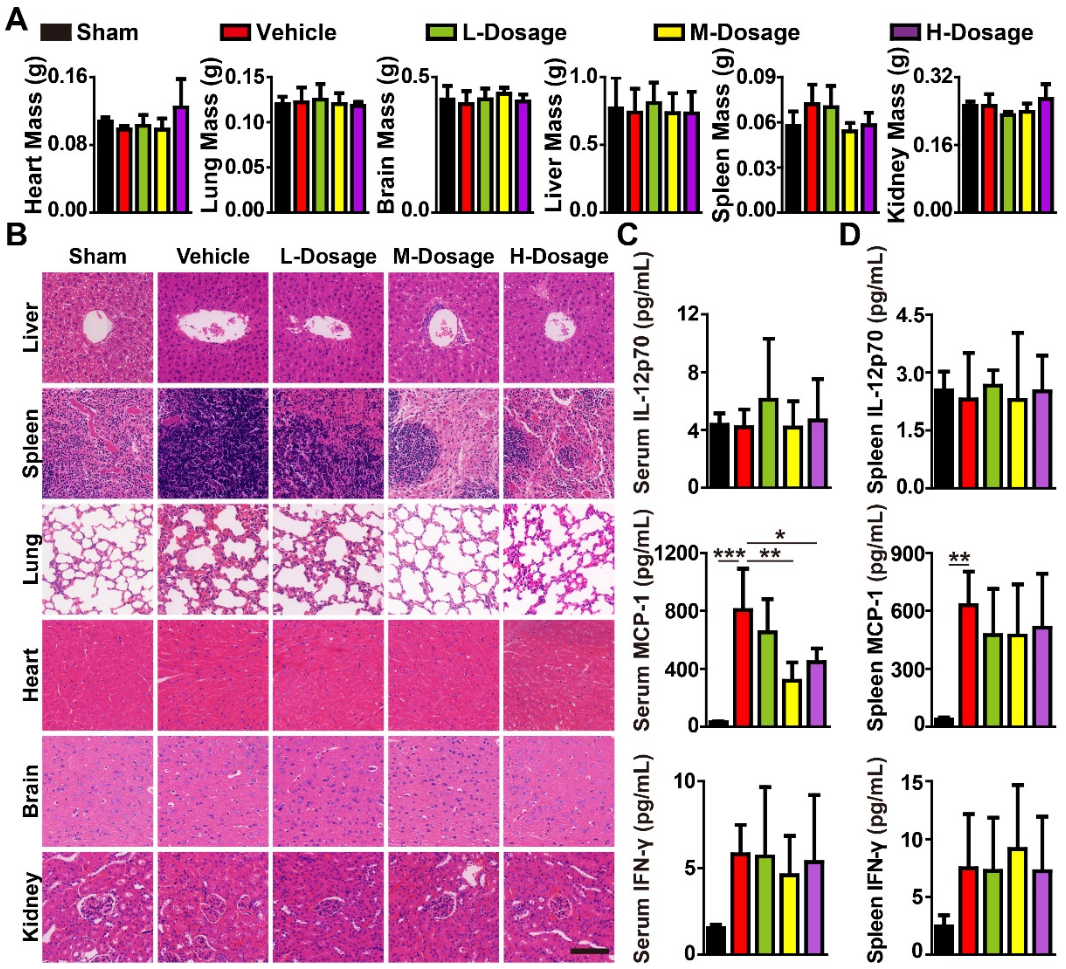

Figure S1. F-AgÅPs mitigate CLP-induced inflammation. (A) Weights of major tissues from the vehicle-treated sham mice and CLP mice treated with vehicle or different doses of F-AgÅPs one time by intravenous injection at 2 h after surgery. Vehicle indicates normal saline (solvent of F-AgÅPs). *n* = 4-5 *per* group. **(B)** Histological analysis of various tissues in (**A**) by H&E staining Scale bar: 100 μm. **(C-D)** Protein level analysis of IL-12p70, MCP-1, and IFN-γ in blood (**C**) and spleen homogenates (**D**) by a CBA inflammation kit. *n* = 5 *per* group. $^{*}P < 0.01$, $^{**}P < 0.01$, $^{***}P < 0.001$.

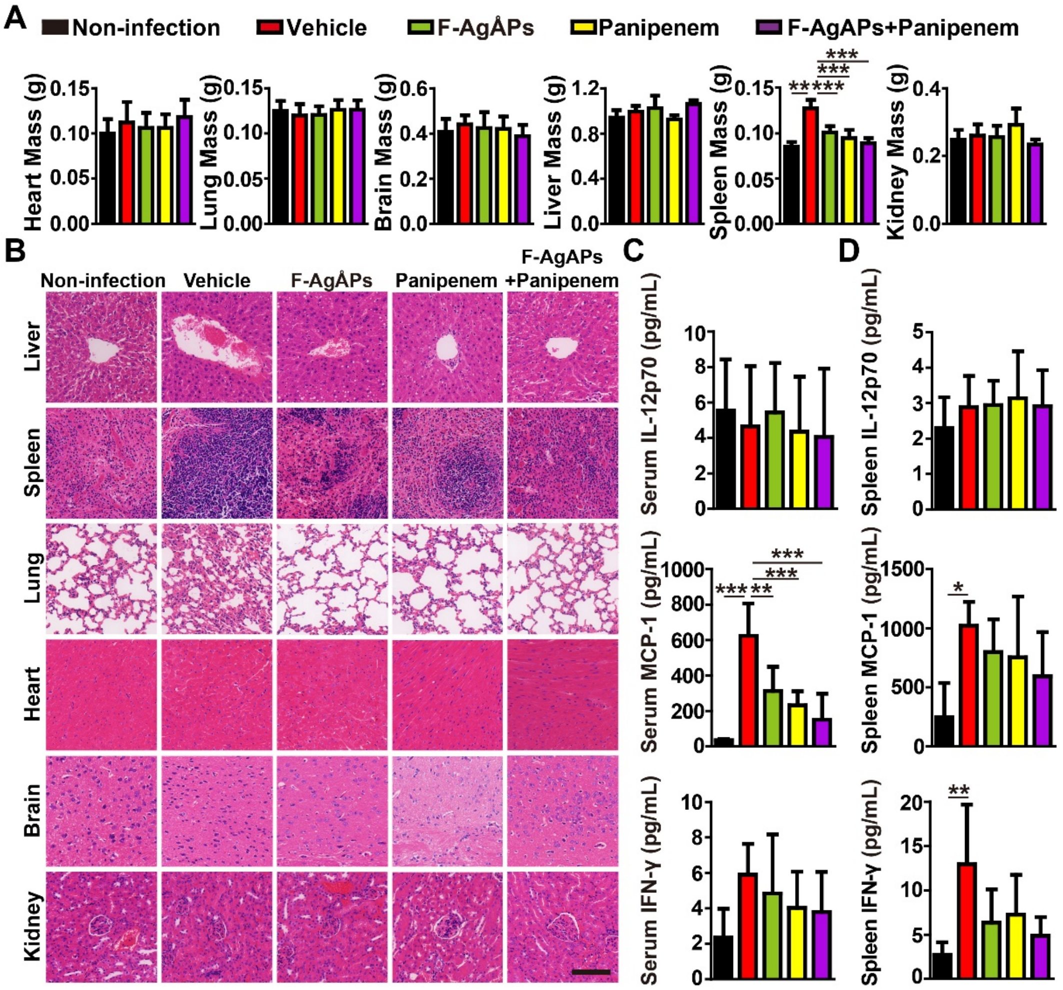

Figure S2. F-AgÅPs mitigate *E. coli* infection-induced inflammation. (A) Weights of major tissues from the vehicle-treated non-infected mice and the carbapenem-sensitive multi-drug resistant ESBL-producing *E. coli*-infected mice receiving three times injections (at 2 h, 24 h, and 48 h after infection) of vehicle, F-AgÅPs, panipenem, or F-AgÅPs + panipenem. Vehicle indicates normal saline (solvent of F-AgÅPs). $n = 5$ *per* group. **(B)** H&E staining images of various tissues in (**A**). Scale bar: 100 μm. **(C-D)** Protein level analysis of IL-12p70, MCP-1, and IFN-γ in blood (**C**) and spleen homogenates (**D**) by a CBA inflammation kit. $n = 5$ *per* group. $^{*}P < 0.01$, $^{**}P < 0.01$, $^{***}P < 0.001$.

Table S1. Effects of F-AgÅPs on hematologic indexes in mice with CLP-induced fatal sepsis.

Hematologic indexes	Sham	Vehicle	L-Dosage	M-Dosage	H-Dosage
White blood cells (WBC) 10^9/L	4.60 ± 1.34	2.16 ± 0.85	1.90 ± 0.57	3.16 ± 1.35	4.84 ± 1.51
Red blood cells (RBC) 10^{12}/L	6.45 ± 1.32	8.47 ± 0.96	7.42 ± 0.57	8.74 ± 0.38	7.37 ± 1.41
Hemoglobin (HGB) g/L	97.80 ± 21.27	125.00± 10.77	116.80 ± 16.16	128.40 ± 5.03	112.60 ± 15.83
Platelets (PLT) 10^9/L	391.00 ± 140.66	396.40 ± 138.43	493.40 ± 215.06	468.60 ± 136.37	578.00 ± 139.02
Neutrophil (NEUT) %	11.72 ± 0.88	27.28 ± 7.67	22.18 ± 5.87	15.92 ± 5.26	14.12 ± 2.72
Lymphocytes (LYMPH) %	87.18 ± 0.81	38.00 ± 4.84	62.30 ± 16.11	70.74 ± 15.38	70.44 ± 5.42
Monocyte (MONO) %	0.72 ± 0.22	33.76 ± 9.40	14.88 ± 11.50	12.82 ± 11.04	15.42 ± 3.79

n = 5 *per* group. Data are shown as mean ± SD.

Table S2. Effects of F-AgÅPs on hematologic indexes in mice with severe E. coli bloodstream infection.

Hematologic indexes	Non-infection	Vehicle	F-AgÅPs	Panipenem	F-AgÅPs + Panipenem
White blood cells (WBC) [illegible]	4.58 ± 2.03	2.10 ± 1.03	1.58 ± 0.41	1.80 ± 0.66	1.95 ± 0.37
Red blood cells (RBC) 10^{12}/L	9.19 ± 1.05	9.11 ± 1.14	9.39 ± 1.14	9.52 ± 0.73	9.54 ± 1.38
Hemoglobin (HGB) g/L	137.50 ± 14.40	137.39 ± 17.10	143.7 ± 14.72	146.00 ± 14.80	144.83 ± 19.88
Platelets (PLT) 10^{9}/L	554.00 ± 211.35	341.57 ± 109.68	351.83 ± 60.76	366.18 ± 70.05	332.83 ± 75.33
Neutrophil (NEUT) %	7.27 ± 1.29	18.87 ± 6.35	10.37 ± 1.80	9.43 ± 2.23	6.48 ± 2.10
Lymphocytes (LYMPH) %	91.05 ± 2.99	75.74 ± 4.58	82.22 ± 2.63	84.78 ± 2.37	87.30 ± 4.10
Monocyte (MONO) %	0.87 ± 1.49	3.80 ± 2.75	5.17 ± 1.21	3.48 ± 1.45	4.05 ± 1.76

n = 6-7 *per* group. Data are shown as mean ± SD.

Table S3. Effects of F-AgÅPs on liver and kidney function indicators in mice with severe E. coli bloodstream infection.

Indicators	Non-infection	Vehicle	F-AgÅPs	Panipenem	F-AgÅPs + Panipenem
TP (g/L)	23.88 ± 3.30	29.20 ± 2.41	26.80 ± 5.35	29.64 ± 1.97	27.95 ± 2.26
ALB (g/L)	12.48 ± 2.01	12.60 ± 1.34	11.80 ± 2.44	13.20 ± 1.09	12.65 ± 1.02
GLB (g/L)	11.40 ± 1.33	16.60 ± 1.86	15.00 ± 2.95	16.44 ± 0.93	15.30 ± 1.28
TB (μmol/L)	0.80 ± 0.24	3.08 ± 0.89	2.00 ± 1.38	1.84 ± 1.44	1.15 ± 0.10
DB (μmol/L)	0.40 ± 0.14	2.00 ± 0.58	1.24 ± 0.91	1.08 ± 0.86	0.60 ± 0.00
TBA (μmol/L)	2.76 ± 0.86	5.84 ± 1.76	5.00 ± 2.07	2.76 ± 1.35	2.05 ± 1.06
ALT (U/L)	13.92 ± 2.48	260.44 ± 150.04	90.20 ± 26.56	42.60 ± 31.60	37.65 ± 11.70
AST (U/L)	66.1 ± 15.46	722.08 ± 273.75	403.12 ± 120.39	205.08 ± 152.54	156.20 ± 30.12
BUN (mmol/L)	5.61 ± 1.12	11.52 ± 3.74	7.78 ± 2.90	5.80 ± 1.61	5.01 ± 0.79
SCr (μ mol/L)	14.44 ± 1.56	16.88 ± 1.84	14.92 ± 1.07	14.20 ± 1.47	13.55 ± 2.58
UA (μmol/L)	126.04 ± 69.70	115.68 ± 69.53	143.04 ± 54.36	223.80 ± 67.34	213.70 ± 55.76

TP: total protein; ALB: albumin; GLB: globulin; TB: total bilirubin; DB: direct bilirubin; TBA: total bile acid; ALT: alanine aminotransferase; AST: aspartate aminotransferase; BUN: blood urea nitrogen; SCr: serum creatinine; UA: uric acid. n = 4-5 *per* group. Data are shown as mean ± SD.

Table S4. Primer sequences for qRT-PCR.

Gene	Forward (5’-3’)	Reverse (5’-3’)
Il-1α	CGAAGACTACAGTTCTGCCATT	GACGTTTCAGAGGTTCTCAGAG
Il-1β	GAAATGCCACCTTTTGACAGTG	TGGATGCTCTCATCAGGACAG
Il-6	TAGTCCTTCCTACCCCAATTTCC	TTGGTCCTTAGCCACTCCTTC
Tnf-α	TGAACTTCGGGGTGATCGGTC	CACTTG GTGGTTTGCTACGACG
Il-10	GCTCTTACTGACTGGCATGAG	CGCAGCTCTAGGAGCATGTG
Gapdh	CACCATGGAGAAGGCCGGGG	GACGGACACATTGGGGGTAG
